# Explaining Variation in Parents' and Their Children's Stress During COVID-19 Lockdowns

**DOI:** 10.3389/fpsyg.2021.645266

**Published:** 2021-09-09

**Authors:** Theo Toppe, Roman Stengelin, Louisa S. Schmidt, Naiera Amini, Nils Schuhmacher

**Affiliations:** ^1^Department of Comparative Cultural Psychology, Max Planck Institute for Evolutionary Anthropology, Leipzig, Germany; ^2^Leipzig Research Center for Early Child Development, Department of Education, Leipzig University, Leipzig, Germany; ^3^Department of Education, Leipzig University, Leipzig, Germany; ^4^Department of Psychology, University of Münster, Münster, Germany

**Keywords:** COVID-19, lockdown, stress, family, parents, children, cultural values

## Abstract

The coronavirus pandemic poses a substantial threat to people across the globe. In the first half of 2020, governments limited the spread of virus by imposing diverse regulations. These regulations had a particular impact on families as parents had to manage their occupational situation and childcare in parallel. Here, we examine a variation in parents' and children's stress during the lockdowns in the first half of 2020 and detect the correlates of families' stress. Between April and June 2020, we conducted an explorative online survey among *n* = 422 parents of 3- to 10-year-old children residing in 17 countries. Most participants came from Germany (*n* = 274), Iran (*n* = 70), UK (*n* = 23), and USA (*n* = 23). Parents estimated their own stress, the stress of their own children, and various information on potential correlates (e.g., accommodation, family constellation, education, community size, playtime for children, contact with peers, media consumption, and physical activity). Parents also stated personal values regarding openness to change, self-transcendence, self-enhancement, and conservation. The results indicate a substantial variation in the stress levels of families and their diverse reactions to regulations. Media consumption by children commonly increased in comparison to the time before the pandemic. Parents raising both pre-school- and school-aged children were at a particular risk of experiencing stress in response to regulations. Estimated stress and reactions varied with the age of children and the personal values of parents, suggesting that such variables need to be considered when implementing and evaluating regulations and supporting young families in the current and future pandemic.

## Introduction

In the first half of the year 2020, governments across the globe counteracted the spread of the novel coronavirus by enacting regulations, including lockdowns, proclamations for social distancing, home confinements, restrictions on private and public gatherings, and the closures of educational facilities. Although these regulations reduced the spread of the virus (Dehning et al., 2020), they also challenged people's psychosocial well-being (Aghili and Arbabi, 2020; Zhang et al., 2020). People faced drastic effects related to occupation, social relations, and welfare support, increasing their psychosocial stress (Huang and Zhao, 2020; Marazziti et al., 2020; Pierce et al., 2020).

Families comprising young children had to cope with additional burdens: in quarantine, parents had to manage their occupation in times of major economic impediments and had to invest in housekeeping, childcare, and homeschooling in parallel. It is thus no wonder that these circumstances resulted in a particular exposure to psychosocial distress among families (Campbell, 2020; Chung et al., 2020; Janssen et al., 2020; Jiao et al., 2020; Miller et al., 2020; Zhou et al., 2020; Moscardino et al., 2021; Ravens-Sieberer et al., 2021; Volk et al., 2021).

However, despite these strains posed by Covid-19 regulations, the disruption of families' lives varied considerably, leading to variation in parental stress levels (Brown et al., 2020; Janssen et al., 2020; Jentsch and Schnock, 2020). Identifying the sources of such a variation may help to bundle support to families at a particular risk during regulations. So far, empirical data are scarce, and researchers mostly speculate about the reactions of families to such measures (Katz et al., 2020). Consequently, the studies exploring the determinants of stress in families during regulations are much needed to inform governments and non-governmental agencies on combating this challenge. Doing so may help identify constellations that are of a particular risk and recognize the factors that may help buffer and counteract parental and children's stress.

The current study aims to contribute to this agenda by exploring the reactions of families to COVID-19 regulations in 2020 and identifying the correlates of parents' and children's stress. To this end, we conducted an online survey among parents of 3- to 10-year-old children between April 2020 and June 2020. In particular, we targeted participants from Germany, India, Iran, the UK, and the USA. We approached participants residing in these countries as we aimed at a more representative understanding of the variety with which families respond to regulations. However, because we shared an English version of the survey *via* social media, parents from other countries participated in this study as well. Thus, parents residing in 17 countries participated in the survey: Algeria (*n* = 1), American Samoa (*n* = 1), Austria (*n* = 2), Belgium (*n* = 2), Canada (*n* = 3), Colombia (*n* = 1), Denmark (*n* = 1), Finland (*n* = 1), France (*n* = 2), Germany (*n* = 274), India (*n* = 4), Iran (*n* = 70), Iraq (*n* = 2), Spain (*n* = 3), Sweden (*n* = 1), the UK (*n* = 23), and the USA (*n* = 23). We assessed how parents estimated their own as well as the psychosocial stress of their children during the regulations.

To assess which factors may have contributed to stress levels in parents and children, we assessed diverse predictors such as household characteristics and socioeconomic variables. This included the information on socioeconomic variables, formal education of parents, and aspects of accommodation of families (i.e., number of rooms and access to garden) as these have been linked to the stress in people during the current pandemic (Agberotimi et al., 2020; Ali et al., 2020; Atchison et al., 2020; Jay et al., 2020; Rehman et al., 2021; Volk et al., 2021).

The number of children in a household may have also affected the stress response of parents and children to regulations. For example, siblings may have offered social support that only children would have lacked otherwise. On the other hand, a higher number of children in a household may have exacerbated parental stress due to an increased demand for care and attention. This may have been particularly relevant in constellations in which parents had to supervise children of different ages in parallel. While preschool-aged children and toddlers need constant supervision and primary care, school-aged children demand assistance in homeschooling activities and managing their (digital) peer contact. Likely, the *combination* of differential demands in both age groups (pre-school- vs. school-aged children) elicited a particular stress to parents due to the dual burden of supervising children with largely differential demands.

Further, we assessed parents' personal values to better understand the variation in families' stress levels within and across countries (Bavel et al., 2020). In the current study, we assessed the values on a household level to gain a detailed understanding of the effect of personal values during the pandemic. To this end, we utilized Schwartz's personal values. Schwartz assumes 10 basic values to be universally relevant to humans across the globe (see Supplementary Table 1; Schwartz and Bilsky, 1990; Schwartz, 1992), which are aligned on four superordinate scales: openness to change, conservation, self-enhancement, and self-transcendence. The prioritization of these values, however, varies across individuals and cultural contexts. In general, Schwartz's personal values represent different motivations helping individuals cope with their eco-social environment (Schwartz, 2012) and might thus have shaped how individuals and families dealt with Covid-19 regulations.

For example, variation in how parents approached novel situations (i.e., *openness to change*) may have been associated with their stress during the lockdown: parents valuing openness might have been more flexible to adjust to the novel situation and consequentially may have experienced less stress. Potential links between personal values emphasizing *conservation* (i.e., order, self-restriction, preservation of the past, and conformity) and families' stress during regulations are less conclusive. The degree to which people valued conservation may have aligned with higher stress levels as routines were challenged and spontaneity was needed to navigate novel situations. On the other hand, parents emphasizing conservation may have also been more willing to adhere to regulations and may have felt less intimidated by social restrictions. We further assumed higher stress levels among parents scoring high on *self-enhancement* as they should have been more likely to perceive the lockdown as an impediment of their autonomy (Bavel et al., 2020). In line with this assumption, recent work found a variation in individualism, both within and across societies, being associated with the adherence to pandemic prevention measures (Maaravi et al., 2021). Societies emphasizing individualism over collectivism were more likely to oppose such measures, which may account for increased case rates and more stress experience during a pandemic (see also Kim et al., 2016, on the role of individualism in the response of people to the Ebola epidemic). Parents scoring high on *self-transcendence* may have accepted and implemented regulations with relative ease by outweighing individual needs in favor of the common good. On the other hand, these individuals may have faced particular psychosocial stress as they are prone to being concerned about the well-being of others.

Furthermore, parents reported the quantity of time they had spent with their children in direct interaction and play activities, their demands for homeschooling their children, children's media consumption, digital contact with peers and relatives, physical activity, and the maintenance of the daily routines of children. In addition, parents reported their age, gender, whether they worked from home at the moment of participation and gave information on community sizes and their country of residence.

We also considered specific regulations (e.g., closure of educational facilities) and their duration as these factors have been found to impact families' stress substantially (Brooks et al., 2020; Golberstein et al., 2020; Roccella, 2020; Röhr et al., 2020). To complement such subjective ratings on the regulations, we added data of the Oxford Covid-19 Government Response Tracker to our analyses (Hale et al., 2020). This tracker systematically indicates policy responses to the COVID-19 pandemic on a stringency score ranging from 0 to 100 (with higher values indicating stronger regulations).

Finally, we added Hofstede's individualism score of national culture from Hofstede's national culture survey (Hofstede, 2021) as a proxy for eventual differences in the stress levels of families across countries. We did so to better account for cross-country differences in meaningful psychological properties and the following previous work relating individualism to the spread of COVID-19 and other pandemic situations (Maaravi et al., 2021).

We added both the stringency score of the Oxford COVID-19 Government Response Tracker and Hofstede's individualism score to our analyses following suggestions by anonymous reviewers.

## Methods

### Participants

A total of *n* = 422 parents from 17 countries participated in this study. Concerning our sample size, we refrained from conducting an *a priori* power calculation as no previous studies were available based on which we could estimate expectable effect sizes. However, we aimed at collecting full data sets from at least *n* = 200 participants because—as a rule of thumb—there should be at least 10 cases per predictor in regression models comprising several predictors (e.g., Wilson Van Voorhis and Morgan, 2007).

We collected the data between April 29, 2020 and June 7, 2020, across different targeted countries (i.e., USA, UK, India, Iran, and Germany). We focused on these countries as they were all affected by the pandemic but vary in their cultural orientations (e.g., Hofstede, 2011), allowing us to gain more generalizable data than surveys focusing on single countries only. Further, many (middle-class) families in these countries could participate in the survey given unrestricted internet access. Notably, there were also participants residing in other countries than the five targeted ones (7.58%), who were included in the analyses. Further, the stringency of the policy responses to the COVID-19 pandemic (*M* = 62.92, SD = 7.93, range = 38.34–86.55) and Hofstede's individualism score (*M* = 64.44, SD = 14.02, range = 13–91) substantially varied across the countries of residence of participants'.

We advertised a German, English, and Farsi version of the survey *via* mailing lists and postings on social media platforms. Further, we asked professionals working in daycare centers, schools, and welfare organizations to share the link to the survey. We utilized *formr* (Arslan et al., 2018) to create each online survey version. Surveys were translated from English by native speakers and double-checked by fluent speakers. Conceptual disagreements between translations occurred rarely and were solved through discussion until we achieved mutual agreement.

Participants provided informed consent by confirming that they participated voluntarily, understood the objectives of this study, and knew that they could withdraw from participation at any time. Participants did not receive any incentives besides their scientific contribution. We did not obtain any information, which could be traced back to individual participants. The study was approved by the Max Planck Institute for Evolutionary Anthropology Child Subjects Committee following legal requirements in Germany.

Table 1 offers a description of the sample characteristics. While we approached both fathers and mothers of young children, most participants were female (89.00%). Most participants were married, were from Germany, and held University degrees at bachelor's or master's levels. Participants were aged between 23 and 65 years (*M* = 38.23, SD = 5.50) and mostly lived together with one or two children in their household (*M* = 1.83, SD = 0.77). The majority of participants lived in urban communities with more than 1,000,000 inhabitants. Around half of the parents (53.83%) reported raising pre-school-aged children, whereas 64.83% reported raising school-aged children. Thus, 18.66% of the parents raised both pre-school- and school-aged children in parallel. About half of the parents worked from their homes at the time in which the survey was conducted.

**Table 1:** Sample description.

**Variable**	**Value**	**Variable**	**Value**	**Variable**	**Value**
**Basic information**		**Community**		**Accommodation**	
Age in years M (SD)	38.23 (5.5)	Country of residence%^a^		Access to private garden%	76.79
Gender%		Germany	65.55	Balcony%	55.22
Female	89.00	Iran	16.75	Access to a park or forest%	89.23
Male	10.77	United Kingdom	5.50	Pets%	34.21
Other	0.24	United States	5.50		
Single parents%	9.81	Community size (No. of citizens)%		Regulations%	
Relationship status%		< 500	2.87	Restrictions on leaving the accommodation	39.95
Married	76.56	< 1500	3.59	Closure of educational facilities	96.65
In a relationship	14.83	< 5,000	7.18	Restrictions on public transport	48.56
Single	4.55	< 20,000	8.85	Extent of regulations	
Divorced	3.83	< 100,000	7.66	Not leaving the house at all	1.44
Widowed	0.24	< 500,000	6.46	Leaving the house for essential activities	44.26
		< 1,000,000	7.18	Leaving the house less than normally	52.63
Educational degree%		> 1,000,000	56.22	Leaving the house as usual	1.67
Secondary degree	13.98			Quarantine duration	
A level	1.42	Household constellation M (SD)		No restrictions	0.72
Bachelor degree	26.78	No. of adults in the household	2.11 (0.75)	Less than one week	0.00
Master degree	51.12	No. of children in the household	1.82 (0.75)	One week	0.24
PhD	6.64			Two weeks	0.24
		Accommodation M (SD)		Three weeks	17.70
Home office%	58.13	No. of bedrooms	6.36 (2.47)	More than three weeks	81.10
		Housing condition%			
		Owner-occupied house	35.17	Stringency score	62.94 (7.93)
		Owner-occupied apartment	17.46		
		Rented house	10.29	Individualism score	64.44 (14.02)
		Rented apartment	37.08		

a *Here, we listed the residences that included >5% of the sample only*.

### Materials and Procedures

Our study consisted of an online survey containing different scales as outlined in the following parameters. Parents needed ~30 min for participation. After giving informed consent, parents reported their age, gender, relationship status, sociodemographic information, community size, country of residence, and current regulations related to the COVID-19 pandemic. Further, they indicated the descriptions of their accommodation, the number of children in their household, and whether children would typically attend a school or daycare institution.

Participants described their current daily life in the following sections, focusing on their school- and pre-school-aged children (assessed separately). Parents provided this information on all children in the respective age group (pre-school- vs. school-aged children). Parents who reported to have two or more children attending school were asked to merge their impressions considering both these children to describe their current daily life. The same applied to pre-school-aged children.

The descriptions of everyday lives of families comprised the information on direct interactions of children with their parents, siblings, peers, and relatives living outside the household and the information on digital communication, daily routines, physical activity, media consumption, and homeschooling of children. Also, participants reported on how harmful and beneficial they perceived current regulations affecting them. We gathered this information using Likert scales.

In the next section, parents indicated their stress levels on the Parental Stress Scale (Zelman and Ferro, 2018), an established scale in clinical psychology. Here, we used the subscales on parental rewards and stressors. Parents indicated their agreement with statements on different aspects of their stress level during the last 2 weeks on a five-point Likert scale (e.g., “Having children leaves little time and flexibility in my life” and “I am happy in my role as a parent”). Internal consistencies were acceptable (α_Reward_ = 0.68; α_Stress_ = 0.75). Further, parents reported the stress level of their children by indicating their agreement with the statement, “Compared to before the pandemic, my child(ren)'s current stress level is much higher now” on a five-point Likert scale.

Finally, parents filled out a brief version of the Portrait Values Questionnaire (PVQ; Schwartz, 2003). The PVQ covers 21 items assessing the 10 values assumed by Schwartz's theory of basic values subsumed into 4 main scales: openness to change (O), conservation (C), self-enhancement (SE), and self-transcendence (ST). The PVQ has proven to be a high-quality instrument with adequate sociometric properties (see Schwartz, 2003). Internal consistencies were acceptable (α_O_ = 0.70; α_C_ = 0.70; α_SE_ = 0.70; α_ST_ = 0.63).

We investigated whether the scales measured the same latent constructs across the three translations. Specifically, we sought to establish a weak measurement invariance as we were interested in the relations between our independent variables and the stress level of parents and children across various countries. To this end, we compared a model in which loadings were constrained to be equal across translations with a model in which the loadings of a dimension were estimated freely across translations.

The invariant model was supported for the reward dimension for the Parental Stress Scale (Δχ^2^(4) = 3.77, *p* = 0.44). The model with free loadings significantly outperformed the model with invariant loadings for the stress dimension (Δχ^2^(4) = 10.26, *p* = 0.04). For the PVQ, the metric model was supported for the conservation (Δχ^2^(10) = 16.81, *p* = 0.08), self-enhancement (Δχ^2^(6) = 8.94, *p* = 0.18), and self-transcendence dimension (Δχ^2^(8) = 10.61, *p* = 0.23). The model with free loadings significantly outperformed the model with invariant loadings for the openness dimension (Δχ^2^(10) = 18.72, *p* = 0.04). Thus, by large, a variation in the latent constructs assessed by our scales likely reflects the same individual differences across the English, German, and Farsi version of the questionnaire.

### Data Analysis

First, we provide the descriptive metrics regarding the experiences and daily lives of parents during the pandemic as such data are sparse. We also report explorative *t*-tests for items related to the change since the COVID-19 outbreak, in which we explored the mean against the “no change” values of the respective scale.

Afterwards, we report the results of an explorative inferential analysis. We ran generalized linear mixed models using the *lme4* package (Bates et al., 2017) in *R* (R Core Team, 2018). These exploratory models aimed to identify the predictors of parents' and children's stress (for a list of all predictors and scaling, see Supplementary Table 2). Notably, the stringency score and Hofstede's individualism score were added as Level 2 predictors (i.e., country-level) to explain variability in intercepts across countries; all other variables were Level 1 predictors (i.e., household level). All metric predictors were standardized. To eliminate the inflation of type I errors, we compared the fit of a model comprising all predictors of interest (hereafter: full model) with a model lacking these predictors and consisting of the intercept only (hereafter: null model; see Forstmeier and Schielzeth, 2011). In case of significant full-null model comparisons, we proceeded with the detailed analyses of each predictor. We ran likelihood ratio tests for the detailed analyses comparing full models with reduced models not comprising each predictor. Thus, statistically significant full-null model comparisons were a necessary condition for detailed analyses and served as a gatekeeper reducing the total number of tests.

To rule out multicollinearity between predictors, we calculated variance inflation factors (VIFs; Field, 2005), using the function *vif* of the *car* package (Fox and Weisberg, 2011). When including both the stringency and individualism score, VIFs suggested high multicollinearity between these predictors (note that both variables were coded on a country level). Therefore, we investigated the effect of the individualism score in a separate analysis. That is, we ran a model including the stringency score and interpreted the effects of all predictors. After that, we ran a model comprising the individualism score instead of the stringency score and focused on a statistically significant effect of the individualism scale. None of the VIFs indicated the issues regarding multicollinearity (all VIFs < 5.14).

## Results

### Descriptive Results

**Figure 1:**
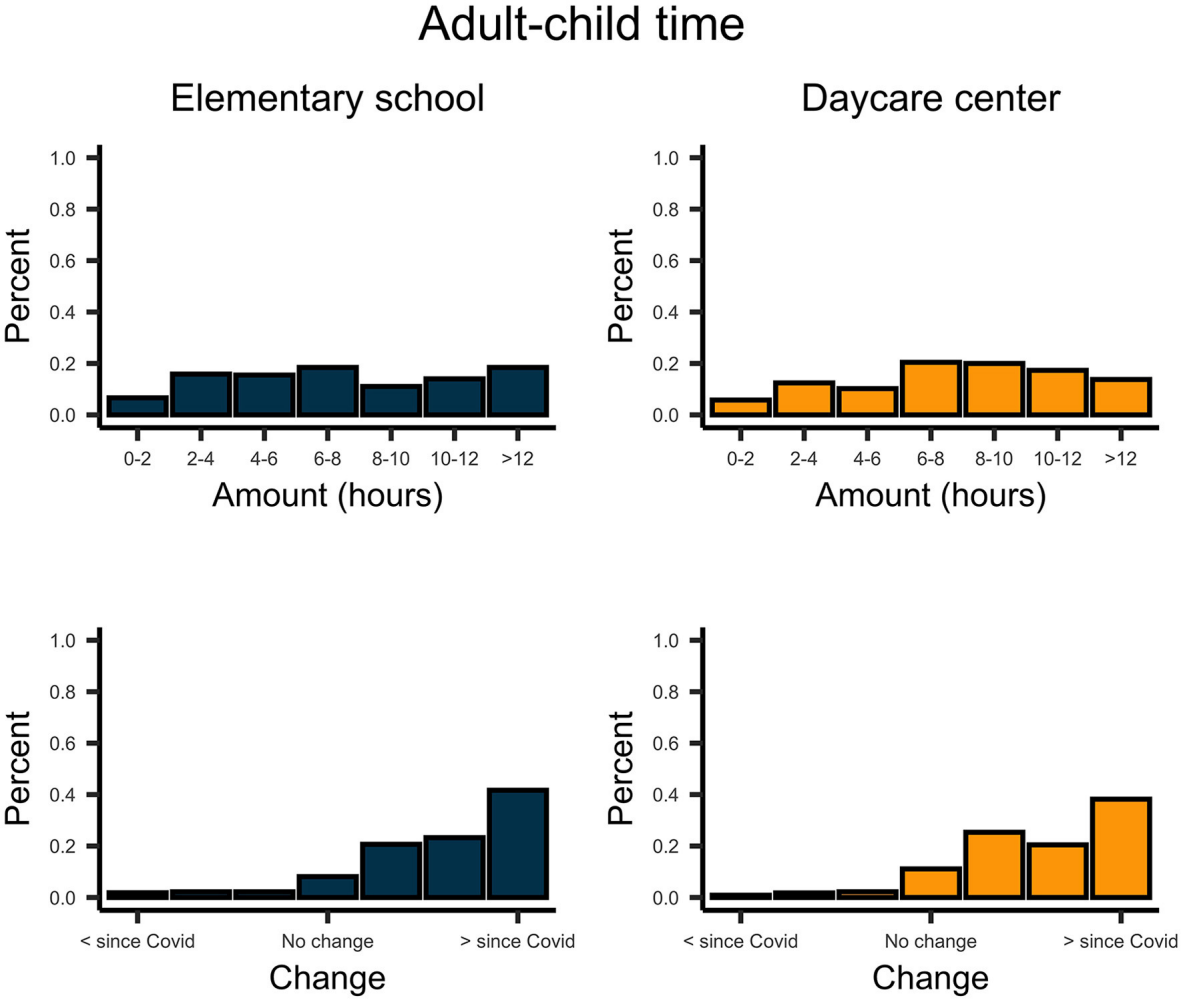
Upper plots indicate the number of hours parents spent with their school- and preschool-aged children. Lower plots indicate the change of this time since the COVID-19 pandemic.

**Figure 2:**
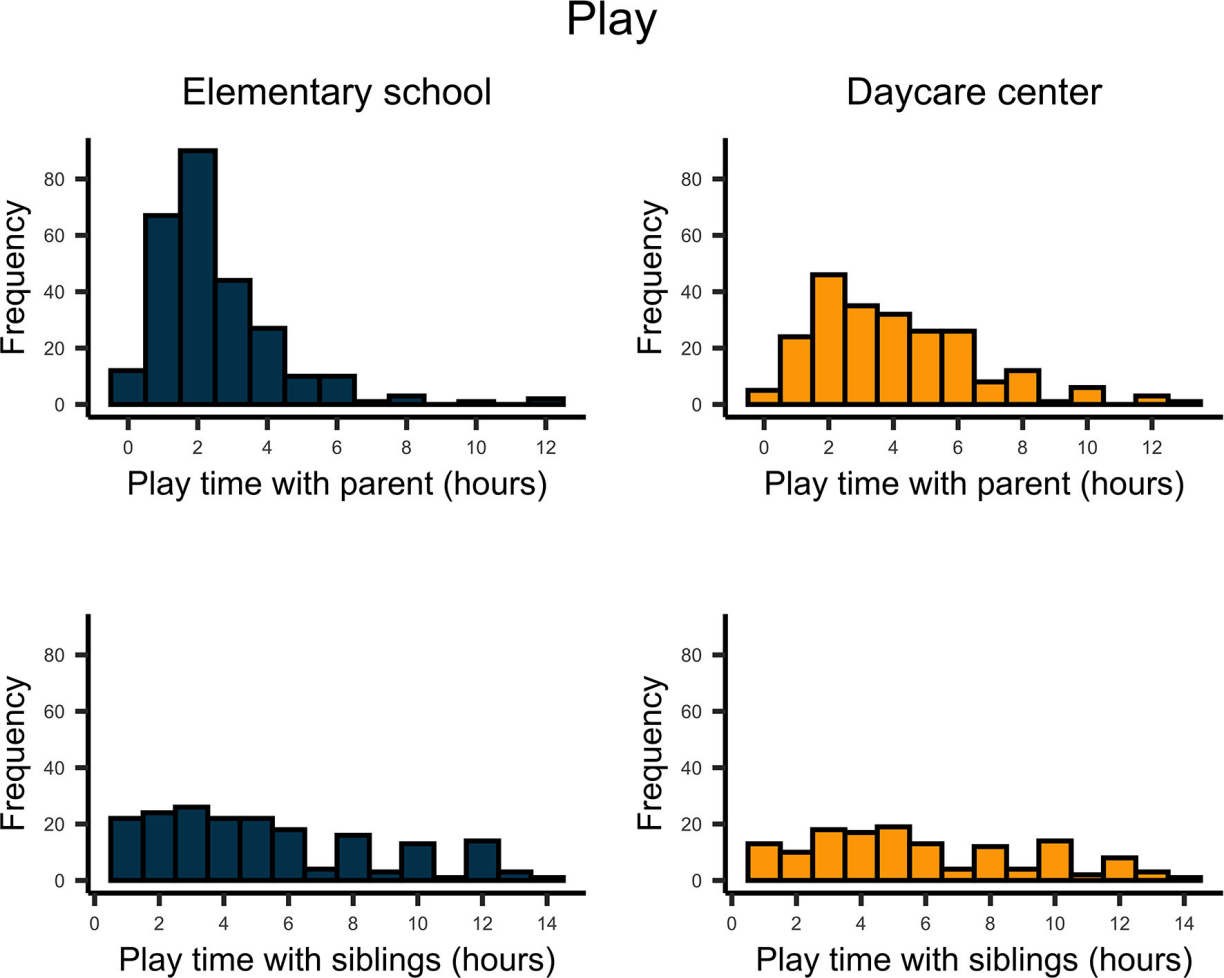
Upper plots indicate the number of hours parents played with their school- and preschool-aged children. Lower plots indicate the time siblings spent for playing.

**Figure 3:**
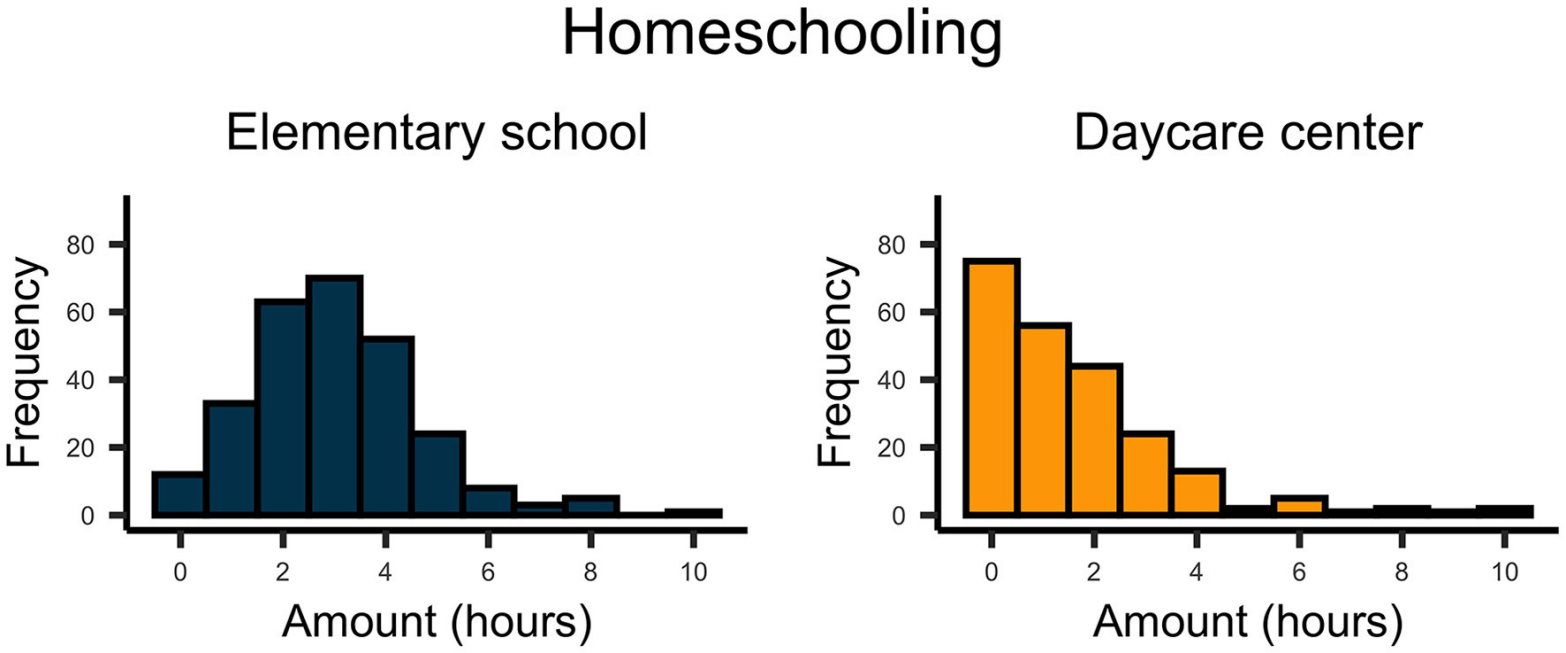
Plots indicate the number of hours parents currently spent for homeschooling with their school- and preschool-aged children.

The time parents directly interacted with their children varied considerably for both school and preschool children (Figure 1). Overall, this adult–child time increased substantially since the beginning of the COVID-19 outbreak for pre-school-, *t*_(224)_ = 19.732, *p* < 0.001, *d* = 1.32, and school-aged children, *t*_(270)_ = 21.335, *p* < 0.001, *d* = 1.30. On average, parents played about 4.0 h with their pre-school-aged children and 2.5 h with their school-aged children (Figure 2). In contrast, parents spent about twice as much time homeschooling their school-aged children (3 hours) than their preschool-aged children (1.5 hours; Figure 3).

**Figure 4:**
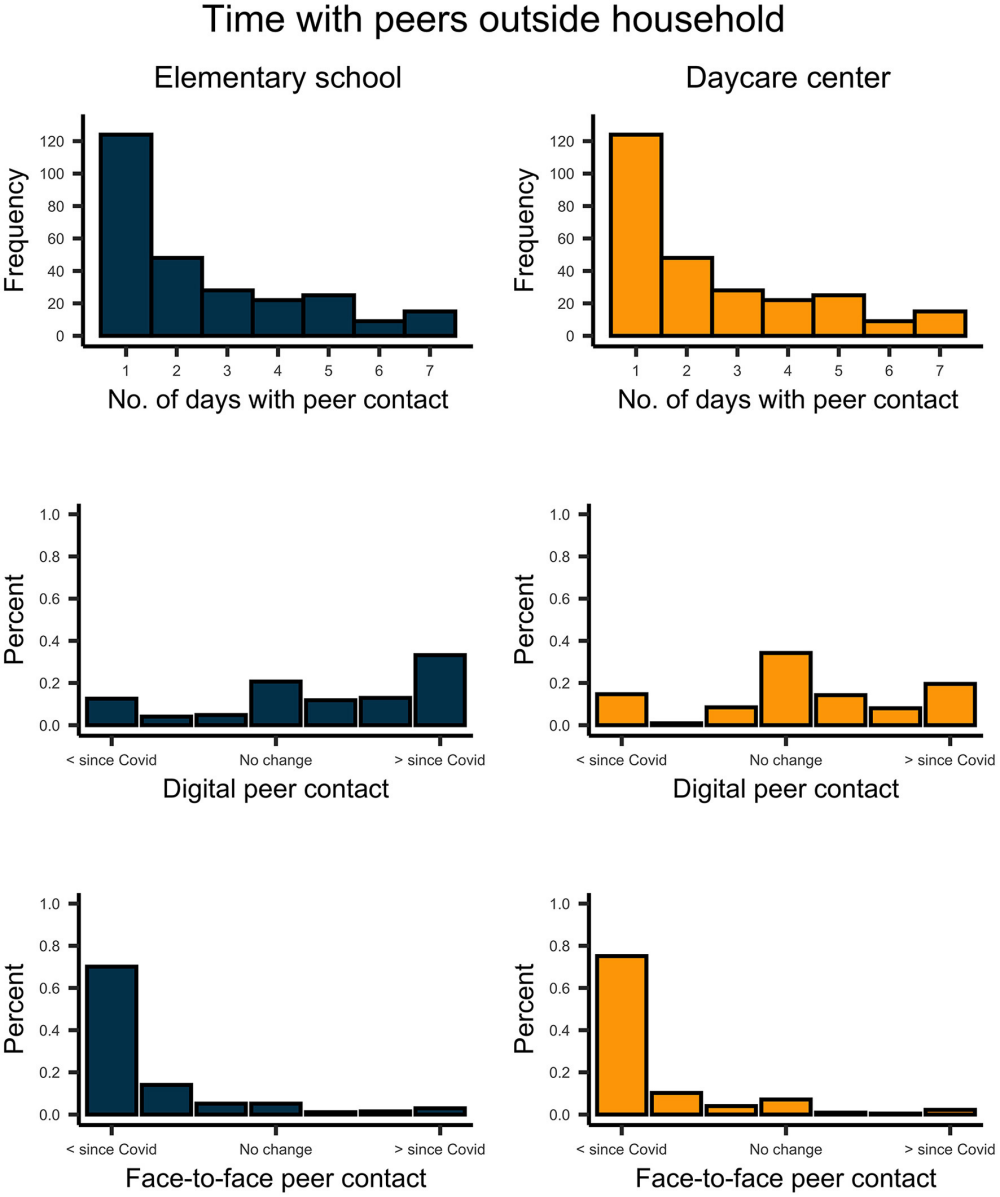
Upper plots indicate the number of days per week on which school- and preschool-aged children had contact with peers living outside their household. Plots in the middle indicate the change of children's digital peer contact since the COVID-19 pandemic. Lower plots indicate the change of children's face-to-face peer contact since the COVID-19 pandemic.

**Figure 5:**
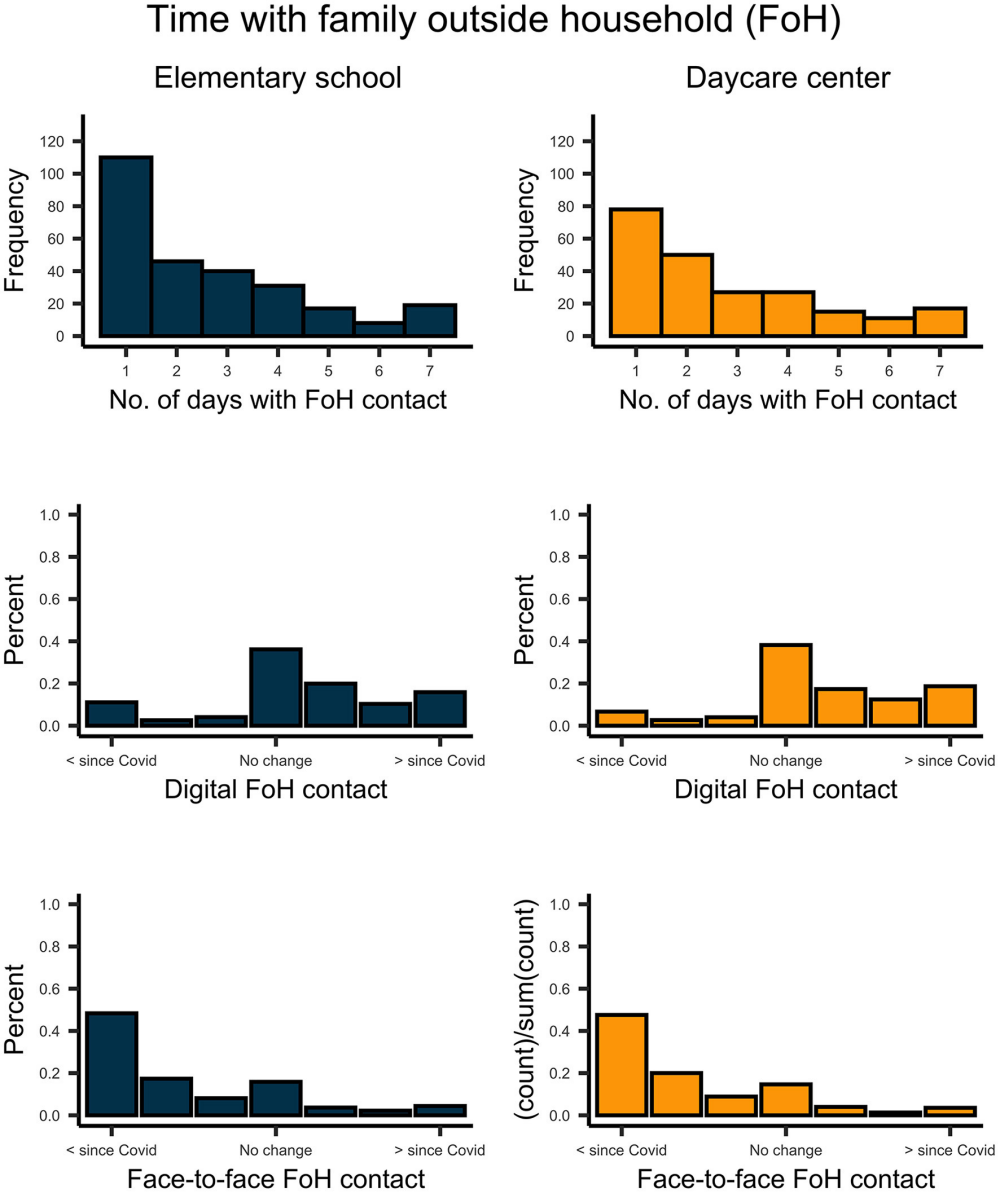
Upper plots indicate the number of days per week on which school- and preschool-aged children had a contact with family members living outside their household. Plots in the middle indicate the change of children's digital contact with family members living outside their household since the COVID-19 pandemic. Lower plots indicate the change of children's face-to-face contact with family members living outside their household since the COVID-19 pandemic.

Face-to-face interactions with peers decreased considerably in response to regulations for both pre-school-, *t*_(224)_ = 28.454, *p* < 0.001, *d* = 1.90, and school-aged children, *t*_(270)_ = 27.212, *p* < 0.001, *d* = 1.65 (Figure 4). On average, children communicated with peers *via* digital means once a week. For school-aged children, digital communication with peers increased markedly since the lockdown, *t*_(270)_ = 6.944, *p* < 0.001, *d* = 0.42. This trend was less pronounced but still significant for pre-school-aged children, *t*_(224)_ = 2.757, *p* = 0.006, *d* = 0.18. A similar pattern was observed for digital contact with family members living outside the household (Figure 5). Here, there was a decrease in face-to-face contact since the lockdown for pre-school-, *t*_(224)_ = 16.339, *p* < 0.001, *d* = 1.09, and school-aged children, *t*_(270)_ = 16.114, *p* < 0.001, *d* = 0.98. For most households, the frequency of digital communication with family relatives remained unaffected by the lockdown. However, we still find a significant increase for preschoolers', *t*_(224)_= 6.366, *p* < 0.001, *d* = 0.42, and schoolers', *t*_(270)_= 4.362, *p* < 0.001, *d* = 0.26, digital contact to family members living outside their household.

**Figure 6:**
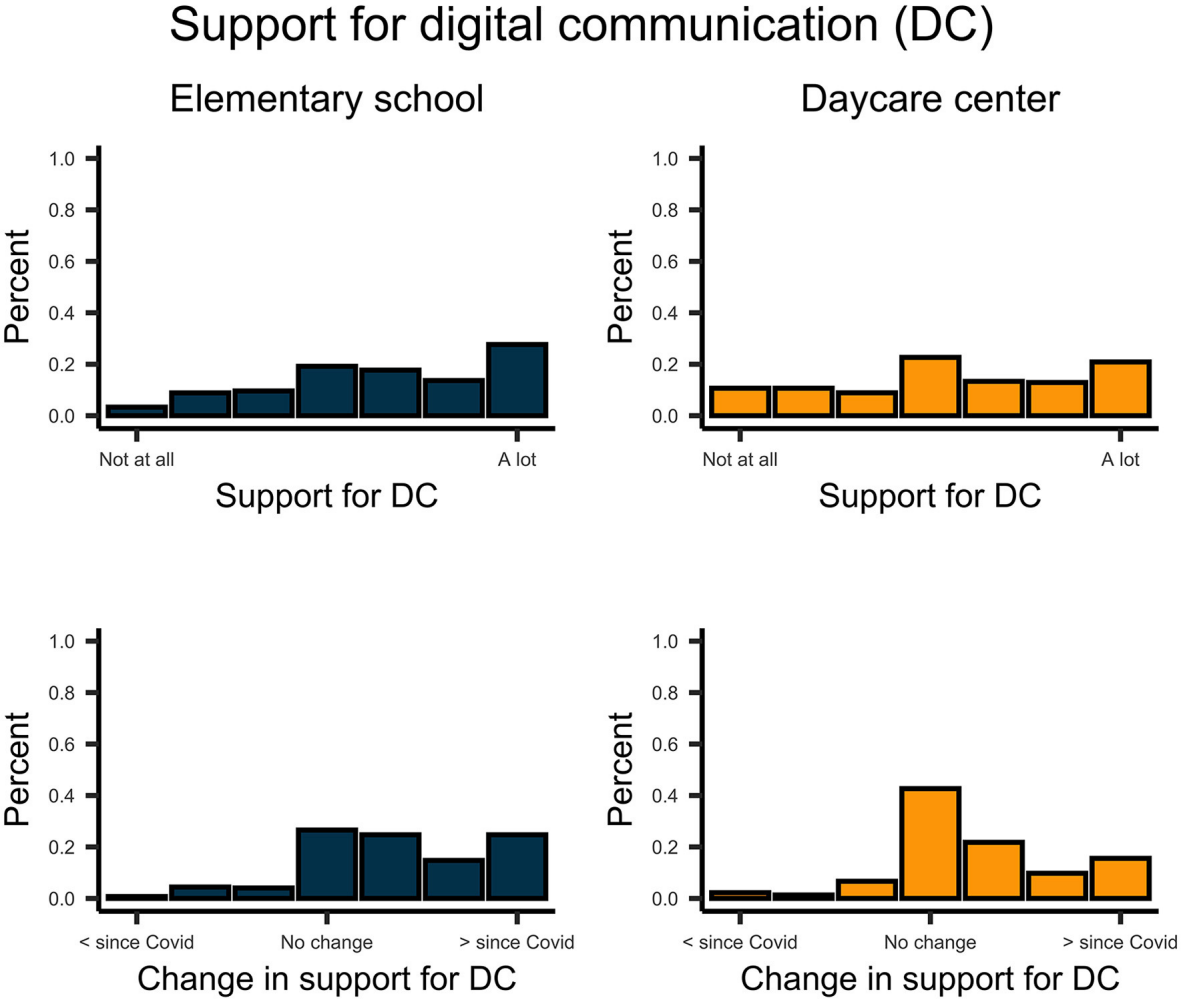
Upper plots indicate the support of parents for digital communication to school- and preschool-aged children. Lower plots indicate the change of this support since the COVID-19 pandemic.

**Figure 7:**
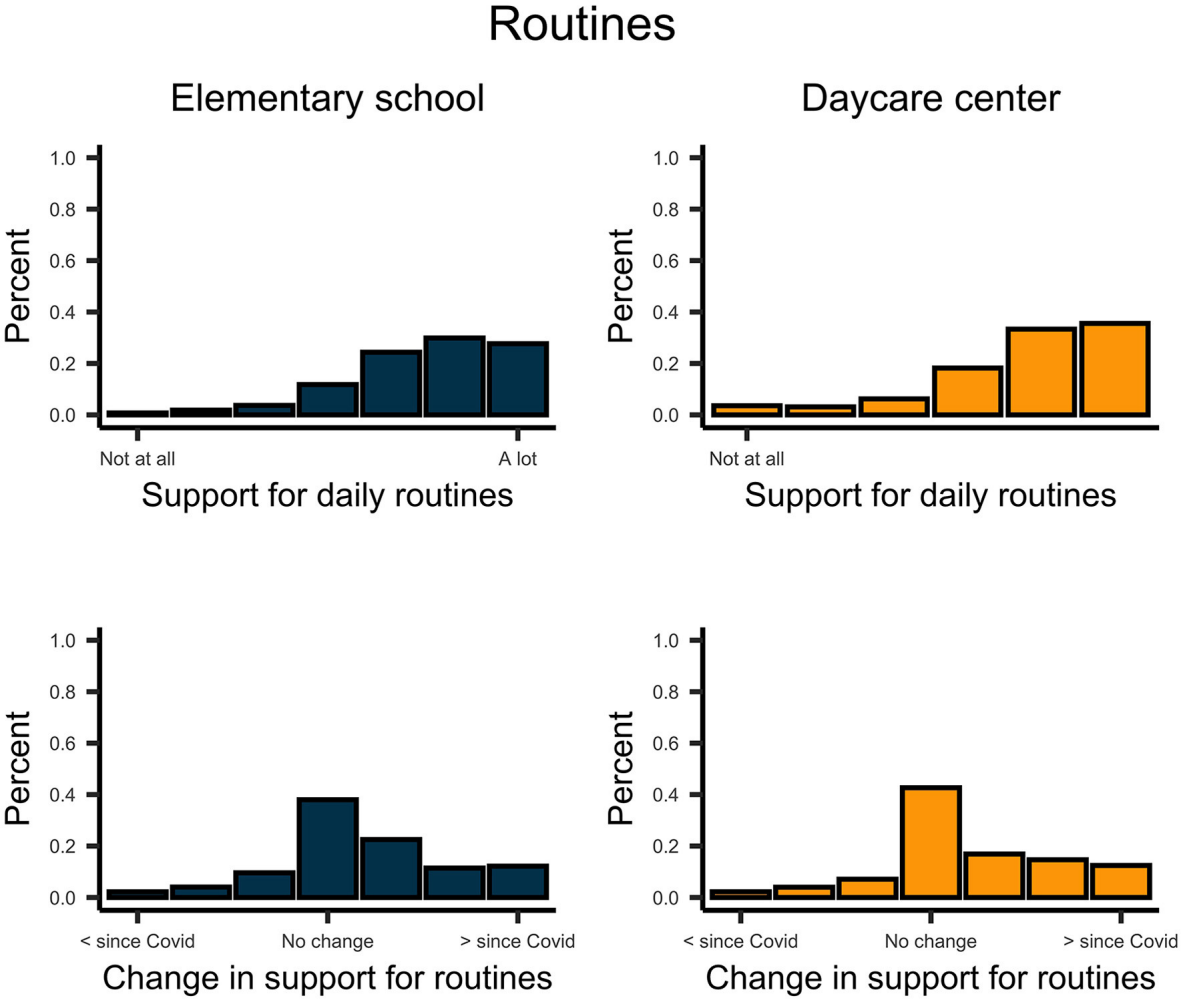
Upper plots indicate the support of parents for the routines of school- and preschool-aged children. Lower plots indicate the change of this support since the COVID-19 pandemic.

**Figure 8:**
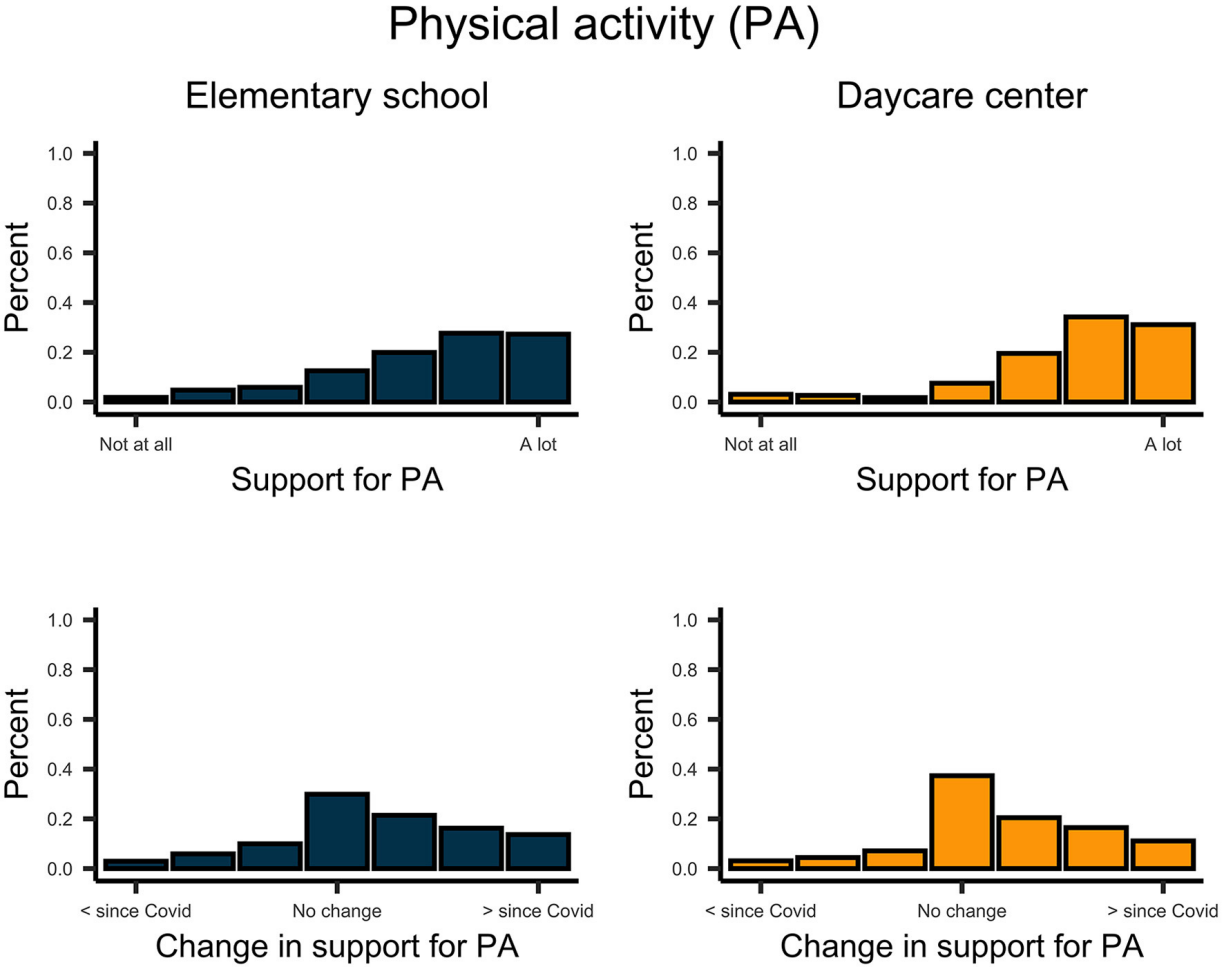
Upper plots indicate the support of parents for the physical activity in school- and preschool-aged children. Lower plots indicate the change of this support since the COVID-19 pandemic.

**Figure 9:**
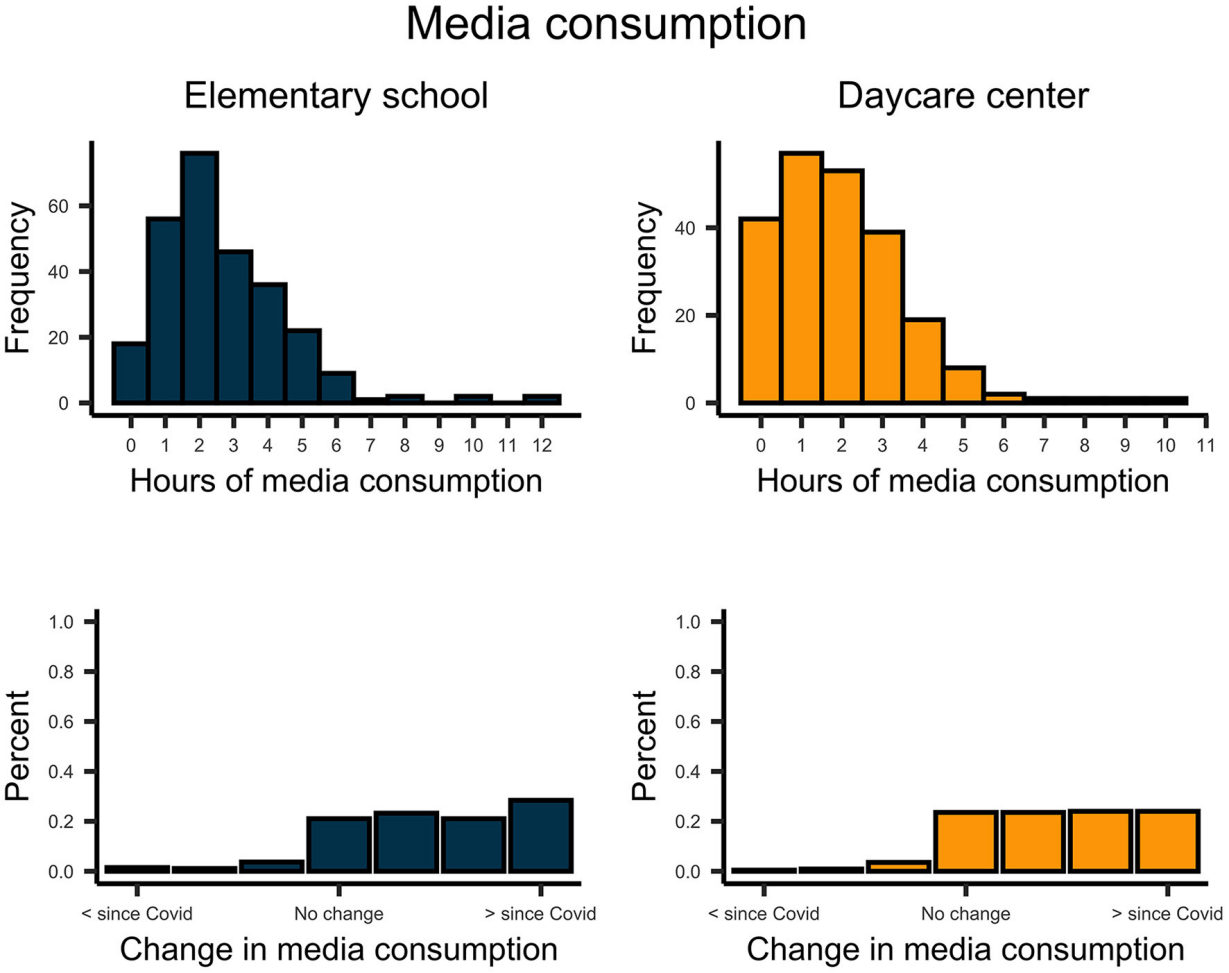
Upper plots indicate the daily number of hours school- and preschool-aged children spent for media consumption. Lower plots indicate the change in the media consumption by children since the COVID-19 pandemic.

Descriptively, school-aged children reported more support from their parents on digital communication than did pre-school-aged children (Figure 6). However, support and permission for digital communication by parents increased for both school children, *t*_(270)_ = 12.958, *p* < 0.001, *d* = 0.79, and pre-school children, *t*_(224)_ = 8.009, *p* < 0.001, *d* = 0.53. In general, parents facilitated daily routines and physical activity for their children (Figures 7, 8). This support significantly increased since the implementation of regulations for both school children, *t*_routines(270)_ = 6.854, *p* < 0.001, *d* = 0.42; *t*_activity(270)_ = 6.985, *p* < 0.001, *d* = 0.42; and pre-school children *t*_routines(224)_ = 6.664, *p* < 0.001, *d* = 0.44, *t*_activity(224)_ = 6.467, *p* < 0.001, *d* = 0.43. Both school- and pre-school-aged children spent about 2.5 h per day with media consumption (Figure 9). Parents reported a substantial increase in media consumption by children since the lockdown, for school-aged children, *t*_(270)_ = 16.809, *p* < 0.001, *d* = 1.02, and for pre-school-aged children, *t*_(224)_ = 16.233, *p* < 0.001, *d* = 1.08.

**Figure 10:**
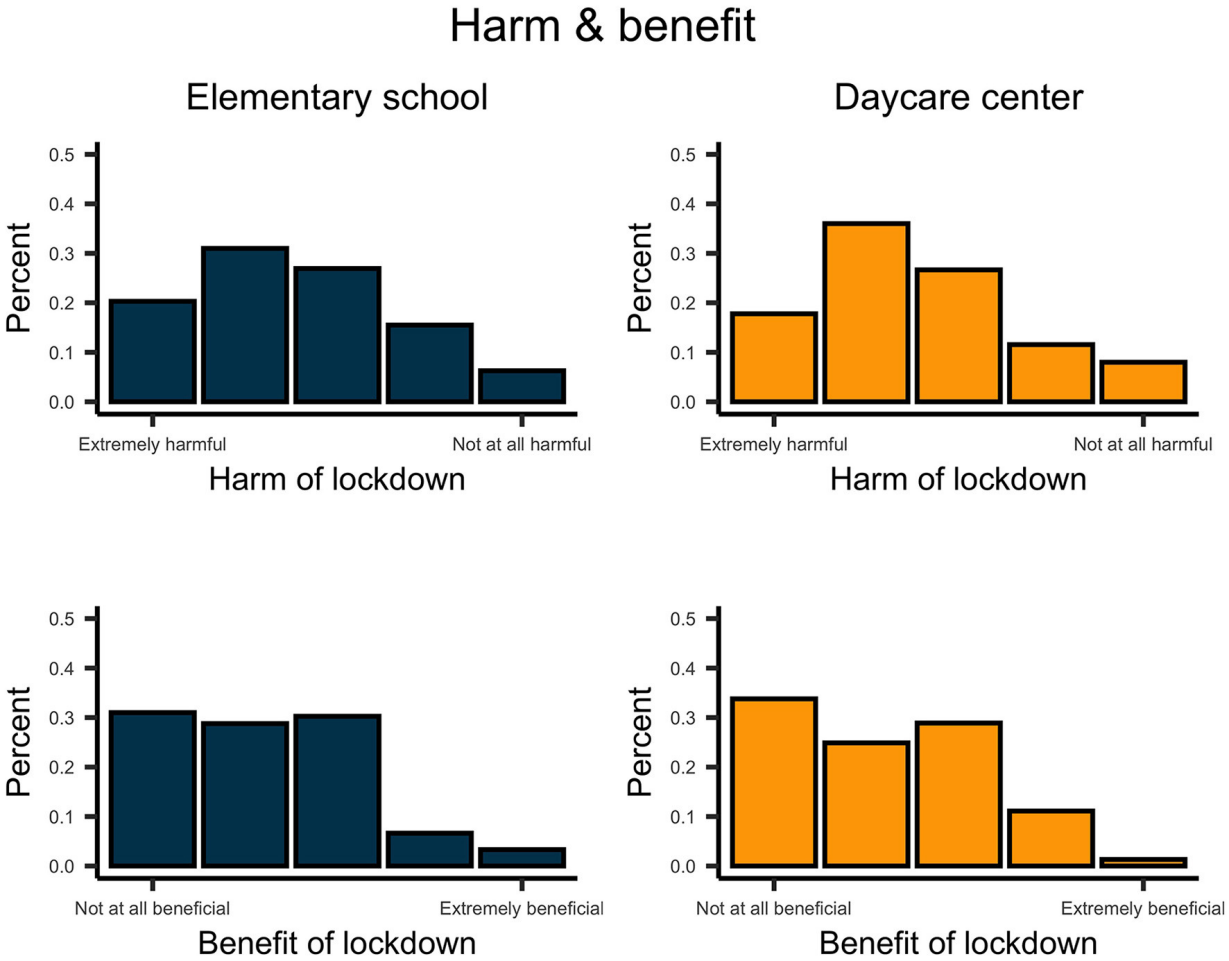
Upper plots indicate how harmful parents perceive the lockdown for their school- and preschool-aged children. Lower plots indicate how beneficial parents perceive the lockdown for their school- and preschool-aged children.

**Figure 11:**
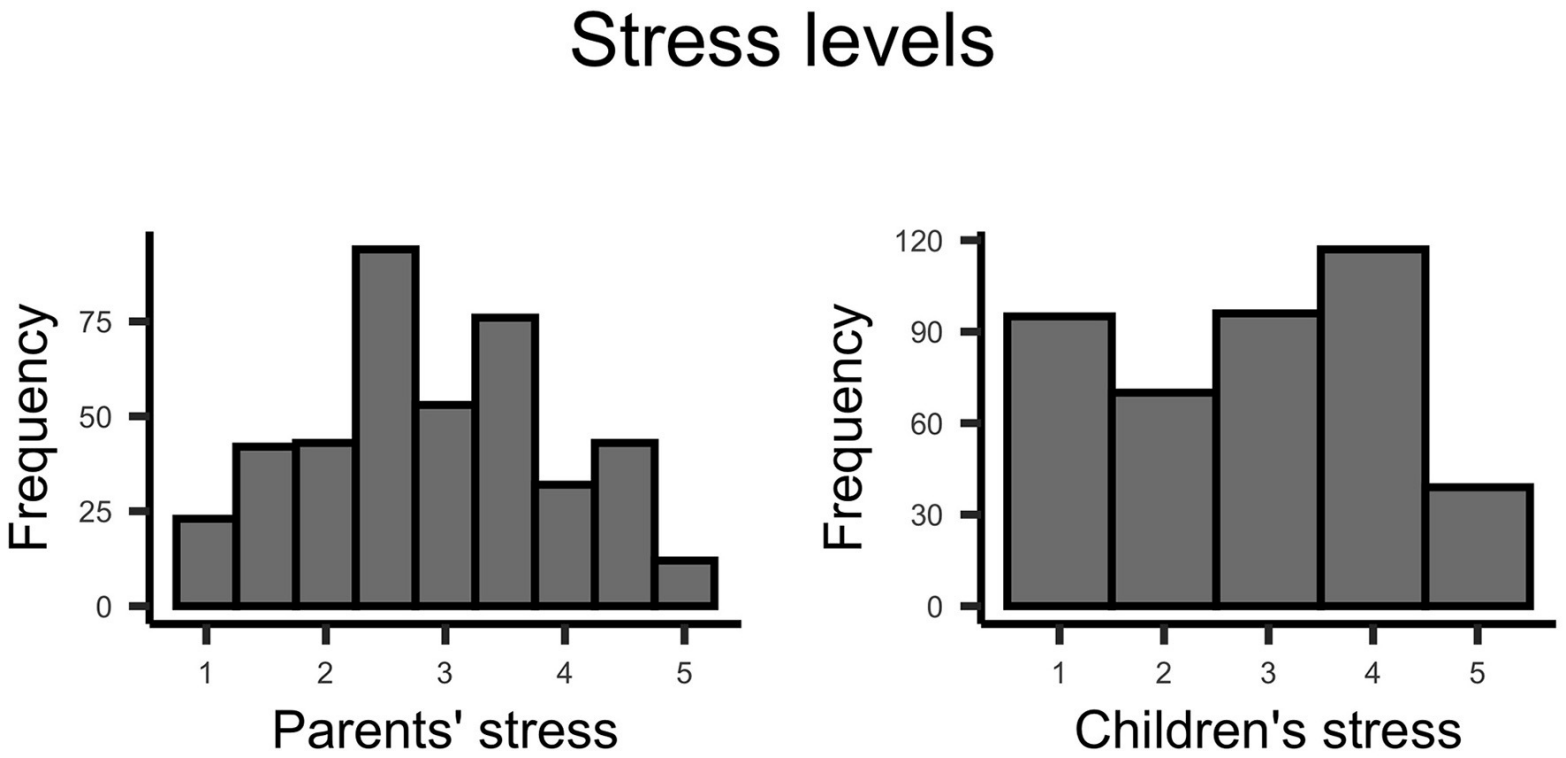
Plots indicate the stress levels of parents and children when taking the survey.

Mostly, parents perceived the lockdown as harmful and non-beneficial for the development of their children (Figure 10). The stress of parents and their judgment regarding the stress of their children varied considerably across parents (Figure 11); however, on average, parents indicated medium to high stress levels.

### Inferential Analyses

We were interested in determining the effects of various predictors on parents' and children's stress during the lockdown. To this end, we ran separate analyses for the data of parents of daycare children (age 3–5 years) and parents of school-aged children (age 5–9 years) to account for the differential demands (i.e., supervision needed, mobility, and child autonomy). It is noted that 18.5% of the parents reported living together with both pre-school- and school-aged children. We included this subsample in both analyses.

In each of the two models, we explored parents' or children's stress using a set of predictors (see Table 2, first column). To account for a systematic variation in the predictors and outcomes across countries, models comprised the country of residence of participants as a random intercept. To underline the robustness of the current findings, we accounted for a large proportion of participants living in Germany by running identical analyses with German participants only.

**Table 2:** Outcomes for inferential models.

	**Parents' stress**				**Children's stress**			
	**Preschool-aged children**		**School-aged children**		**Preschool-aged children**		**School-aged children**	
**Full-null model comparison**	***χ***^**2**^**(38) =69.902**, ***p*****= 0.001**		***χ***^**2**^**(38) = 79.382**, ***p*****< 0.001**		**χ^**2**^39)= 87.163**, ***p*****< 0.001**		**χ^**2**^(39)= 62.026**, ***p*****= 0.011**	
	***estimate***	***S.E.***	***estimate***	***S.E.***	***Estimate***	***S.E.***	***estimate***	***S.E.***
Age	0.003	0.075	−0.105	0.073	0.072	0.100	0.116	0.094
Gender (ref: Male)								
Female	0.058	0.219	0.249	0.230	−0.382	0.293	−0.554	0.297
Other	1.124	0.980	−0.036	0.984	0.064	1.317	0.666	1.270
Home office	0.020	0.145	−0.031	0.136	0.201	0.195	0.233	0.174
Education (ref: Secondary)								
A level	0.097*	**0.442**	0.080	0.980	0.584^*^	**0.592**	−0.962	1.265
Bachelor	0.059^*^	**0.261**	−0.173	0.218	−0.268^*^	**0.350**	0.149	0.276
Master	0.291^*^	**0.224**	−0.014	0.192	−0.497^*^	**0.301**	−0.098	0.246
PhD	0.972^*^	**0.319**	0.533	0.308	−0.970^*^	**0.438**	−0.366	0.395
Single parent	0.170	0.305	0.239	0.206	0.456	0.409	0.016	0.264
Parents' stress	**–**	**–**	–	–	0.254^*^	**0.097**	0.316^*^	**0.091**
Openness	−0.011	0.076	–0.154^*^	**0.071**	−0.014	0.102	−0.102	0.091
Self-enhancement	0.045	0.078	0.153^*^	**0.076**	−0.017	0.105	0.058	0.098
Self-transcendence	0.148^*^	**0.077**	0.033	0.076	−0.330^*^	**0.103**	−0.068	0.097
Conservation	−0.055	0.079	–0.182^*^	**0.073**	0.058	0.105	−0.027	0.095
Community size (ref: < 500)								
< 1500	0.279	0.743	0.215	0.428	−0.595	0.995	−0.087	0.544
< 5000	0.018	0.591	0.140	0.391	−0.567	0.792	−0.024	0.494
< 20.000	−0.112	0.588	−0.254	0.379	−0.089	0.788	0.399	0.479
< 100.000	0.494	0.591	−0.397	0405	−0.379	0.792	0.190	0.511
< 500.000	0504	0.600	0.528	0.434	−0.836	0.805	−0.700	0.554
< 1.000.000	0.315	0.601	0.211	0.415	−0.852	0.804	−0.344	0.524
1.000.000+	0.054	0.561	0.167	0.344	−0.424	0.751	−0.170	0.435
Number of rooms	0.045	0.081	−0.009	0.082	−0.208^*^	**0.109**	−0.180	0.103
Garden	−0.318^*^	**0.167**	−0.044	0.175	0.052	0.226	0.045	0.223
Restrictions	−0.058	0.144	−0.138	0.137	−0.204	0.193	0.264	0.174
Stringency score (Level 2)	0.179	0.121	0.037	0.184	0.099	0.159	−0.354^*^	**0.157**
Individualism score (Level 2)^a^	0.197^*^	**0.111**	0.221	0.140	0.142	0.149	0.095	0.165
Constellation of children	0.405^*^	**0.185**	0.494^*^	**0.149**	−0.084	0.250	−0.232	0.196
Number of children	0.019	0.084	0.098	0.071	0.132	0.112	0.113	0.091
Parent-child time	0.090	0.079	−0.075	0.080	0.178	0.106	0.073	0.103
Change in parent-child time	0.109	0.074	0.215^*^	**0.074**	−0.235^*^	**0.100**	−0.068	0.095
Homeschooling	−0.124	0.070	0.012	0.066	−0.039	0.095	0.066	0.084
Digital peer contact	0.146^*^	**0.071**	–0.146^*^	**0.066**	0.035	0.097	0.046	0.086
Support for digital contact	−0.024	0.081	−0.126	0.078	0.062	0.109	0.020	0.099
Change in support for digital contact	−0.032	0.082	0.019	0.075	0.045	0.109	0.008	0.096
Support for physical activity	−0.241^*^	**0.096**	−0.074	0.080	−0.003	0.130	−0.061	0.102
Change in support for physical activity	0.103	0.084	0.042	0.076	0.283^*^	**0.112**	0.270^*^	**0.097**
Support for routines	0.115	0.085	−0.004	0.079	−0.225^*^	**0.114**	−0.088	0.100
Change in support of routines	−0.009	0.086	−0.015	0.078	0.215	0.115	−0.107	0.100
Media consumption	−0.101	0.092	−0.007	0.085	0.151	0.123	0.009	0.107
Change in media consumption	0.143	0.080	0.064	0.074	0.224^*^	**0.107**	0.220^*^	**0.094**
Model determination (Marginal)	0.248		0.178		0.285		0.193	

*SE; boldly printed estimates and SEs marked with an asterisk indicate a significant pairwise comparison with p < 0.05. Model determination indicates marginal effects of all fixed effects*.

a *For the individualism score, estimates and SEs of the separate model are reported here*.

#### Parents' Stress

A full-null model comparison indicated that the combined set of predictors had a statistically significant effect on stress levels among parents of daycare children, ${\text{χ}}_{\text{full}-\text{null}}^{2}$(38) = 69.902, *p* < 0.001 (Table 2). Likelihood ratio tests revealed a statistically significant effect of parental education, ${\text{χ}}_{\text{education}}^{2}$(4) = 14.390, *p* = 0.006. To explore this effect further, we ran multiple Tukey *post hoc* comparisons using the package *multicomp* (Hothorn et al., 2008). These tests suggested that parents holding a PhD reported particularly high stress levels compared to other parents and that the stress level of parents of all other educational levels did not vary substantially (see Supplementary Table 3). For the personal values of parents, the model revealed a positive association between parents' stress and their values on self-transcendence, ${\text{χ}}_{\text{ST}}^{2}$(1) = 4.189, *p* = 0.041. Parents from households with access to a garden reported less stress than parents living in households without garden access, ${\text{χ}}_{\text{garden}}^{2}$(1) = 4.214, *p* = 0.040. Parents raising both pre-school- and school-aged children (*M* = 3.25, SD = 0.92) reported higher stress levels than those raising pre-school-aged children only (*M* = 2.78, SD = 1.10), ${\text{χ}}_{\text{constellation of children}}^{2}$(1) = 6.288, *p* = 0.012. Children's digital peer contact was positively associated with the stress level of parents, ${\text{χ}}_{\text{digital peer contact}}^{2}$(1) = 5.399, *p* = 0.020. Finally, we found a negative link between the support parents provided for the physical activity of their pre-school-aged children and their own level of stress, ${\text{χ}}_{\text{physical activity}}^{2}$(1) = 7.951, *p* = 0.005. That is, parents who offered more support for the physical activity of their pre-school-aged children reported feeling less stressed. A separate model revealed that Hofstede's individualism score predicted higher stress levels among parents, ${\text{χ}}_{\text{individualsm}}^{2}$(1) = 4.047, *p* = 0.044.

We found an overall effect of the variables for parents of school-aged children, ${\text{χ}}_{\text{full}-\text{null}}^{2}$(38) = 79.382, *p* < 0.001. Pairwise model comparisons suggested negative associations between parents' stress and their values on openness to change, ${\text{χ}}_{\text{O}}^{2}$(1) = 5.155, *p* = 0.023, and conservation, ${\text{χ}}_{\text{C}}^{2}$(1) = 7.296, *p* = 0.007, and a positive association between stress and self-enhancement, ${\text{χ}}_{\text{SE}}^{2}$(1) = 4.601, *p* = 0.032. Again, the constellation of children was linked to the stress level of parents, ${\text{χ}}_{\text{constellation of children}}^{2}$(1) = 12.462, *p* < 0.001, such that parents of both pre-school- and school-aged children had a higher stress level (*M* = 3.25, SD = 0.92) than parents of school-aged children only (*M* = 2.90, SD = 0.97). The change in time spent between parents and their children was positively linked to parents' stress, ${\text{χ}}_{\text{change in parent}-child\;time}^{2}$(1) = 9.856, *p* = 0.002. The more parents spent time in a direct interaction with their school-aged children, the more stress parents reported. Finally, the more school-aged children contacted their peers digitally, the less stress was reported by parents, ${\text{χ}}_{\text{digital peer contact}}^{2}$(1) = 5.543, *p* = 0.019.

#### Children's Stress

For parents of pre-schoolers, we found a statistically significant effect of the combined set of predictors, ${\text{χ}}_{\text{full}-\text{null}}^{2}$(39) = 87.163, *p* < 0.001. Likelihood ratio tests revealed a statistically significant effect of parental education, ${\text{χ}}_{\text{education}}^{2}$(4) = 11.170, *p* = 0.025. Again, we ran multiple Tukey *post hoc* comparisons using the package *multicomp* (Hothorn et al., 2008) to explore this effect. These tests did not suggest a clear pattern (see Supplementary Table 4). However, an inspection of the estimates suggests that parents holding a lower educational degree report more stress of their children. Parents' stress was positively associated with children's stress, ${\text{χ}}_{\text{parent}{\text{s}}^{\text{′}}\text{ stress}}^{2}$(1) = 7.936, *p* < 0.005. Parental values on self-transcendence were negatively linked to children's stress, ${\text{χ}}_{\text{self}-\text{transcendence}}^{2}$(1) = 11.687, *p* < 0.001, such that children whose parents strongly valued self-transcendence were described as being less stressed. Children living in households with more rooms were described as less stressed, ${\text{χ}}_{\text{number of rooms}}^{2}$(1) = 4.850, *p* = 0.028. The change in time parents spent with their pre-school-aged children since COVID-19 was linked to children's stress, ${\text{χ}}_{\text{change in time spent}}^{2}$(1) = 6.411, *p* = 0.011. The more time parents spent with their children had increased in response to the pandemic and accompanying regulations, the less stressed were children described by their parents. Besides, the more parents supported the routines of their children, the less stress was reported, ${\text{χ}}_{\text{routines}}^{2}$(1) = 5.666, *p* = 0.017. The change in parents' support for children's physical activity since Covid-19 was linked to children's stress, ${\text{χ}}_{\text{change in physical activity}}^{2}$(1) = 7.896, *p*= 0.005. Here, increased support was associated with more stress. Finally, the change in media consumption by children was related to their stress level, ${\text{χ}}_{\text{change in media consumption}}^{2}$(1) = 6.439, *p* = 0.011, with increasing media consumption going along with higher stress levels of children.

For parents of school-aged children, we found an overall effect of the combined set of predictors, ${\text{χ}}_{\text{full}-\text{null}}^{2}$(39) = 62.026, *p* = 0.011. Similar to pre-school-aged children, parents' stress was positively associated with school-aged children's stress, ${\text{χ}}_{\text{parent}{\text{s}}^{\text{′}}\text{ stress}}^{2}$(1) = 13.973, *p* < 0.001. Children's stress was also positively linked to media consumption, ${\text{χ}}_{\text{media consumption}}^{2}$(1) = 6.884, *p* = 0.009. Again, the change in parents' support for children's physical activity since the lockdown was positively associated with children's stress, ${\text{χ}}_{\text{change in physical activity}}^{2}$(1) = 9.812, *p* = 0.002. Finally, the stringency of countries was negatively linked to children's stress ${\text{χ}}_{\text{stringency}}^{2}$(1) = 5.609, *p* = 0.018.

When analyzing the German subsample only, we find a pattern of results that is similar to the results based on the full sample (see Supplementary Table 5).

## Discussion

The coronavirus pandemic had and has drastic effects on the life of young families around the globe. The current study explored the potential correlates of psychosocial stress among parents and their children in response to COVID-19 regulations exhibited in the first half of 2020.

Our study indicates moderate to high levels of stress among parents and their children. Parents perceived the situation as harmful rather than beneficial, with a considerable variation in this evaluation across families. Unsurprisingly, parents spent more time with their children as compared to the time before the pandemic and engaged in more homeschooling. Children engaged in fewer face-to-face interactions with peers and family members living outside their households, which was substituted with an increased emphasis on digital communication. Further, parents reported that their children's media consumption increased substantially compared to the time before the pandemic. This finding on increased media consumption in young children during COVID-19 regulations resonates with recent studies (Feierabend et al., 2020; Hartshorne et al., 2021; Poulain et al., 2021).

To explore variation in parents' and children's stress, we estimated the effect of various variables. Overall, we find (a) mostly different predictors for parents' stress as compared to children's stress and (b) different associations among pre-school- and school-aged children.

Notably, the cross-sectional design of our study does not allow for causal conclusions regarding these associations. The detected associations between families' stress and some variables should not be conceived of as monocausal. This particularly applies to variables subject to situational changes (e.g., changes in media consumption and homeschooling activities). It is most likely that these variables affected parents' and children's stress and, at the same time, were affected by these stress levels. Given the cross-sectional research design, we can only speculate on the directionality of links between personal values and stress. Personal values have likely changed in response to the pandemic and governmental regulations, rendering monocausal interpretations of such associations premature. Discussions regarding the directions of the detected effects remain speculative and require confirmation by longitudinal and experimental studies. We focus below on the most prominent and congruent associations in the current data and reasonable interpretations thereof.

### Parents' Stress

#### Constellation of Children

We found that parents raising both pre-school- and school-aged children were more stressed than those raising children from one of the two age groups only. It appears that the confrontation with a dual load of childcare was particularly stressful for parents. During COVID-19 regulations, parents needed to provide primary care and close supervision for pre-schoolers, whereas school-aged children needed support for homeschooling and (digital) peer interactions. The presence of both types of demands may have enhanced psychosocial stress of parents.

It is important to note that this effect was evident regardless of the absolute number of children in a given household, indicating that the differential demands posed by pre-school- and school-aged children drove the association. Siblings of roughly similar ages may eventually buffer parents' stress as children support each other during homeschooling or play activities. Moreover, parents may supervise children of similar ages in parallel, increasing the efficiency of parenting interventions during regulations.

Governments and social support systems may particularly consider parents raising both pre-school- and school-aged children when deciding on how to allocate support for families. For example, parents raising both school-aged and preschool-aged children may be given priority to make use of limited (i.e., emergency) childcare programs or access to other support.

#### Education

Parents of pre-school-aged children holding a PhD degree reported higher stress levels during regulations as compared to other parents. Given that lower socioeconomic status is typically associated with heightened stress levels and health issues (Chen and Miller, 2013; Brown et al., 2020), this finding appears surprising at first glance as it contradicts previous work, suggesting more stress among families from lower socioeconomic backgrounds during the Covid-19 lockdown (Agberotimi et al., 2020; Ali et al., 2020; Atchison et al., 2020; Jay et al., 2020; Huebener et al., 2021; Rehman et al., 2021; Volk et al., 2021).

Interestingly, this link was only evident among parents of pre-school-aged, but not older children. We suppose this link may indicate that parents with high levels of education and raising younger children may rely on institutionalized childcare more frequently—most urgently if both parents have a full-time or leading position (Petitclerc et al., 2017; Alt et al., 2018). In times of COVID-19 regulations and a lack of institutionalized childcare, these parents may have aimed to work from home while taking care of their children—leading to exceptionally high levels of psychosocial stress. As such, these parents may have been affected most severely by the closing of daycare institutions, leaving them unprepared to manage caregiving and their occupation in parallel.

One may speculate that some of the parents holding a PhD may have been enrolled in jobs linked to medical care. Accordingly, the association of education and parents' stress is in line with the previous work highlighting the necessity for psychosocial support of medical staff at the frontline in combating the pandemic (Galbraith et al., 2020; Zaka et al., 2020). Notably, caregiving duties have been identified as a stressor for medical staff previously (Mo et al., 2020).

Besides, one may assume that parents in the PhD subgroup may commonly be early career scholars in academia who find themselves in vulnerable situations due to short-time contracts and pressure to generate scientific output (e.g., publications and grant funding). The dual load of compensating closed daycare institutions while being in an insecure career phase may have resulted in high levels of psychosocial stress. Notably, scientists have emphasized the need to support early career researchers during the pandemic (Cheng and Song, 2020; Gibson et al., 2020; Termini and Traver, 2020). Furthermore, some of these parents may have been employed in responsible positions in which the dual load of homeschooling and job occupations may have had more severe effects on their stress.

Another explanation holds that these parents may have had more severe concerns regarding the negative consequences of regulations on the development of their children. If so, these parents may have been susceptible to the disruptive nature of social restrictions, leading them to experience high stress levels by themselves. This account may also explain why these parents did not ascribe higher stress levels to their children—as the adverse consequences of regulations may have been unknown to these children at the time. As we did not assess detailed information on the employment of participants, we can only emphasize that more work is needed to examine these associations by focusing on parents employed in these and other high-risk domains.

Surprisingly, the link between education and stress level did not recur among parents of school-aged children. One may assume that parents with higher degrees in the formal education may be better off to master schooling their children at home. However, it has to be noted that the current study did not investigate homeschooling in detail. While it is plausible that parents with higher degrees in formalized education may have advantages to compensate formal schooling, this does not imply that they conceive this situation as less stressful than parents reporting lower degrees in formalized education.

#### Accommodation

We find that access to a private garden was associated with lower parental stress. Having a garden may be particularly beneficial when families are restricted in mobility (i.e., public parks, sport facilities, and playing grounds). Having a safe environment outside may reduce the urge for close supervision among parents and, thus, provide parents (and children) much needed degrees of freedom during lockdowns. Furthermore, spending time outside might have also reduced the stress levels of parents more directly due to enjoying nature and engaging in recreational gardening activities (Hilbert, 2020). This link also resonates with our finding on children's stress and the number of rooms available in their households (see below).

Of course, possessing a private garden may also be indicative of a higher level of wealth, which may buffer against stress during the pandemic (see also Agberotimi et al., 2020; Ali et al., 2020; Atchison et al., 2020; Jay et al., 2020; Rehman et al., 2021; Volk et al., 2021).

#### Personal Values and Individualism Score

Our investigation revealed different links between the personal values of parents, Hofstede's individualism score, and stress levels. As outlined above, it is important to bear in mind that the current study design does not allow us to identify the causality of this association. While Schwartz' personal values target participants' trait-like (rather than state-like) values (Roccas et al., 2002; Parks-Leduc et al., 2015), significant events such as the COVID-19 pandemic have likely altered the personal values of parents. Stressful experiences might result in substantial disruptions of personal values. As such, causal interpretations of the links between parents' stress and personal values should be made with caution.

For parents of school-aged children, the pattern seems to be straightforward. Higher emphasis on self-enhancement went along with higher stress levels. It is likely that the increased demand for childcare disrupted parents' goal achievement (e.g., job success) and that this was particularly stressful for parents valuing such goals. Alternatively, this link may indicate that parents suffering from higher stress were more sensitive to the disruptive potential of the pandemic situation on their autonomy. Parents being open to change and valuing conservation reported being stressed to lower degrees. Parents emphasizing openness to novel situations might cope better with everyday life changes during the lockdown and may have been more optimistic. For parents valuing conservation, it might have been easier to accept and adapt to measures counteracting the pandemic. Accordingly, these parents may have been eager to take such steps out of a sense of responsibility for the public (see also Bavel et al., 2020; Gelfand et al., 2021). Again, a reverse effect may have been of relevance: parents experiencing lower levels of stress may have had fewer concerns regarding the pandemic situations, and such optimism may have accentuated their personal values regarding openness to change.

We did not find this pattern for parents of pre-school-aged children. Here, parents emphasizing self-transcendence reported being more stressed. One may assume that directedness to others may have led parents to be particularly concerned with the disruptive effects of the pandemics. Further, our results suggest that cultural individualism—as indicated by Hofstede's score—was positively associated with stress for parents of pre-school-aged children. Cultural contexts characterized by loosely knit social networks might promote parents' stress as parents had to cope with the situation more independent of social or family support. Previous findings also suggest that cultural individualism is associated with more severe consequences of the pandemic (Gelfand et al., 2021).

In general, our findings align with those from transcultural psychiatry, showing significant links between personal value orientations and vulnerability to psychological strain (Heim et al., 2019). Personal values reveal important information to predict the stress reactions of parents to the pandemic. Future research is needed to gain a more comprehensive overview of such relations while considering the causality of such links *via* longitudinal study designs. Particularly studies investigating participants from diverse cultural contexts seem a promising avenue in this arena (Bavel et al., 2020; Katz et al., 2020).

#### Behavioral Adaptations

Across the age groups of both children, we found diverse associations between parents' stress and changes since the COVID-19 regulations. Importantly, all these associations do not allow for causal interpretations but may reflect bidirectional links instead.

The more parents of pre-school-aged children facilitated the physical activity of children, the less stress they reported. One way of interpreting this finding is that fostering the physical activity of pre-schoolers may have been an efficient means to promote the mood of children toward being more balanced, which may have had downstream effects on parents' stress. Partial support for this interpretation stems from the studies finding the physical activity of children negatively related to their stress levels (Martikainen et al., 2013; Rodriguez-Ayllon et al., 2019). Given that regulations disrupted the physical activity of children outside their homes (e.g., limited access to club sport), pre-school children (and their parents) may have suffered from these disruptions most heavily. This interpretation also resonates with the finding that pre-school-aged children were less stressed when living in more spacious accommodations allowing for physical activity without relying on public playgrounds (see below). However, it also seems plausible that less stressed parents were better equipped to promote the physical activity of their pre-school-aged children.

School-aged children's contact with peers *via* digital means was linked with parents' stress, such that the facilitation of digital peer contact was associated with lower stress levels. We propose two interpretations for this finding: firstly, parents with lower stress levels may be better equipped to support their children's digital peer contact, which requires high levels of adult supervision and technical know-how. Alternatively, substituting children's in-person contact with their peers through digital means may present an efficient coping mechanism allowing parents to reduce psychosocial stress. Given the increasing importance of peer contact for the psychosocial well-being of children throughout school-age (Rubin et al., 2005, 2007), we argue that encouraging children's digital peer contact offers an important means to substitute social disruptions and stabilize psychosocial well-being.

Interestingly, this association was reversed for parents of pre-school-aged children. Parents facilitating more digital peer contact among pre-schoolers reported higher stress levels. On one hand, stressed parents might have aimed to reduce their strain by promoting digital peer contact. On the other hand, the development of self-regulation and autonomy of children might have driven this effect. That is, school-aged children may have benefitted from their increasing competency to navigate digital media to communicate with peers. Here, media use by children may have buffered parental stress efficiently. Younger children lacking such competence and autonomy may have required more supervision by their parents to engage in digital peer communication, promoting their stress levels.

Irrespective of all these interpretations, our data imply that children's digital peer contact is associated with the stress levels of parents. One potential implication of this is that media competencies of parents could be actively promoted to use school-aged children's digital peer contact as an effective coping mechanism. Furthermore, parents could be equipped with hands-on services allowing for children's digital communication. Providing families with intuitive and secure services to ensure a digital communication between peers might present an effective means to ensure social exchange between children and reduce parents' stress. Eventually, such services might also be helpful for parents of pre-school-aged children as these have more difficulties with the handling of digital communication tools.

### Children's Stress

We found a positive association between parents' own stress and their pre-school- and school-aged children's stress. Parents reporting higher stress tended to report higher stress of their children. Because the reports on both parents' and children's stress were obtained from parents, it stands to reason that those parents suffering from more severe stress may have also ascribed this stress to their children. To counteract this effect, we suggest assessing children's stress directly through self-report in future studies (see Limitations).

This methodological concern notwithstanding, a genuine link between parents' and children's stress levels is also plausible. That is, stressed parents might not have had the capacity to adequately support their children, thereby increasing their children's stress, and stressed children might have been a strain for their parents. This association suggests that children's and parents' stress should not be seen as a separated phenomenon. Families had to cope with the novel situation as units.

We also found associations between adaptive strategies and children's stress. Firstly, the more children consumed media since the lockdown, the more stress parents ascribed to their children. Secondly, the more parents supported the physical activity of children since the lockdown, the more stress parents ascribed to their children. Again, these associations cannot be interpreted in terms of causal directions, and different interpretations are applicable. For example, parents might have allowed more media consumption and might have encouraged more physical activities as a reaction towards their children's increased stress levels. Regardless of the causal inferences drawn from this data, the current findings highlight the role of these two domains for the stress levels of children. Providing parents with resources such as media services (see above) and sports instructions for the physical exercise of children at home seems advisable in this regard.^1^

#### Pre-School-Aged Children

Pre-school-aged children's stress was associated with the number of rooms in accommodations of families, such that more spacious accommodations went along with less stress. In line with the associations found between parents' stress and the physical activity of children and parents' stress and access of families to a garden (see above), one may assume that pre-school-aged children may require sufficient space for their playing activities. Opportunities to children for playing outside were disrupted drastically during COVID-19 regulations in many countries, suggesting that the home environment presented children's playground during this time. Our data point to the vulnerability of families lacking access to supportive accommodations (e.g., spacious housing or access to a garden), such as those living in urban contexts or families from lower socioeconomic backgrounds. As such, our findings hint toward the importance of public playgrounds, parks, and sports facilities for families' the well-being of families well-being in times of regulations. As such, our findings hint toward the importance of public playgrounds, parks, and sports facilities for families' well-being in times of regulations. Instead, granting temporary controlled access for families with young children may be an efficient tool to reduce the stress levels of children with otherwise limited access to playing grounds and space.

Further, we found a link between pre-school-aged children's stress and parents' support for daily routines. This finding resonates with research documenting the promotive effects of daily routines on the well-being of children (Fiese et al., 2002; Kitsaras et al., 2018). Our data confirm the importance of daily routines for reducing the stress level of pre-schoolers' in response to COVID-19 regulations.

#### School-Aged Children

In addition to the abovementioned effect, our data suggest a negative link between the stringency of the COVID-19 regulation and school-aged children's stress. That is, higher stringency went along with less stress. This finding contradicts our predictions and points to the need for more detailed assessments of both children's stress and regulations. Event-based sampling strategies might be useful to assess the impact of specific regulations on children's stress.

### Limitations

As noted above, the cross-sectional design of the current study hinders firm conclusions about the direction of the detected associations. Longitudinal or experimental studies are needed to understand which coping strategies families used during social distancing regulations, which of them were proven useful, and which of them were maladaptive.

It has to be noted that we did not assess reports of children's stress directly but relied on the evaluation of parents, which were assessed with one item only. We chose this approach as it was more convenient and allowed us to assess a larger sample as parents could complete the survey individually. However, ratings of parents on the psychosocial well-being of children's might not be accurate, particularly so if parents are themselves exposed to increased psychosocial stress in response to regulations—rendering a close monitoring of the well-being of their children challenging. The link between parents' and children's stress emphasizes this methodological limitation. The inclusion of the perspectives of children presents an important avenue for upcoming research and can reveal more detailed effects of the COVID-19 pandemic on their psychological well-being.

One limitation regarding the statistical approach of the current study in combination with its explorative nature is the inclusion of diverse predictors into the models but their interactions were not explored. While such an assessment was beyond the scope of the current investigation, a focus on selective interactions in the current data set may offer fruitful insights to learn more about familiar risk constellations in times of regulations. Also, the analysis of subgroups might reveal interesting insights (e.g., families with children from both age groups). Our data can be retrieved online (osf.io/r84ca/), and we encourage scholars to explore this data and test directed hypotheses using this source.

Further, our data were not optimal for the investigation of differences between countries. That is, we only obtained sufficient participants (*n* > 20) from four countries (i.e., Germany, Iran, UK, and USA; Centre for Multilevel Modelling, 2021). As such, the detected effects of cultural individualism and the stringency of the COVID-19 regulation need to be interpreted with caution.

A final limitation concerns the generalizability of our findings based on sample characteristics. Participants were mostly female and came from wealthy urban areas in Global North countries (i.e., Germany and UK). Also, we did not assess ethnic variability within countries. In consequence, the current findings cannot be easily generalized outside such societies. Firstly, it is important to note that most parents partaking in the current study identified themselves as females even though we aimed to assess both fathers and mothers. Numerous studies have emphasized that many of the burdens posed by the pandemic situations have fallen on mothers (Power, 2020; Forbes et al., 2021; Staniscuaski et al., 2021). This particularly applies to variables subject to situational changes (e.g., changes in media consumption, homeschooling activities) (Staniscuaski et al., 2021). More data on the role of fathers in the pandemic is much needed. Following our cross-cultural approach, inter-individual and societal variation in the involvement of fathers in childcare presents a promising variable in this regard. Secondly, the participants of the current study reported high degrees in formal education and socioeconomic status. We also targeted participants from diverse, non-Western societies to gain a more representative perspective (Henrich et al., 2010). Still, the response rate from parents in Global North countries was much higher. A fundamental challenge of such cross-cultural survey research is that it is difficult to isolate the drivers of country-level variation in study findings. Human societies vary alongside multiple factors, including the importance ascribed to formalized education, monetary wealth, ethnicity, household compositions, and cultural values. Parents' occupation of parents in more industrialized and digitalized contexts may have been easier to adjust to home office settings. Furthermore, the COVID-19 pandemic caused more fundamental problems to people residing in the Global South (e.g., related to nutrition, see Amadasun, 2021). As such, one can assume different effects on families' stress in such regions and should be careful generalizing from our findings without further data.

While the identification of intra-cultural and inter-cultural variation in parents' and children's stress is important to gain a better grasp of the psychological correlates of governmental regulations during the pandemic, more targeted research is needed to include more diverse and globally representative participants. Comparing societies converging in some aspects (i.e., individualism) but not others (i.e., severity of regulations) may be helpful to tease apart the drivers of variation on a country level (Norenzayan and Heine, 2005).

### Conclusion

Our investigation provides explorative insights into the correlates of parents' and children's psychosocial stress throughout the first lockdown phase of the coronavirus pandemic in 2020. We documented that families' stress varied substantially during COVID-19 regulations, pointing to the importance of individual factors and the eco-social contexts surrounding these families.

Regulations were not stressful for all, but most families, and personal values concerning openness, self-enhancement, self-transcendence, and conservation were linked to the reported stress levels. We found parents raising both pre-school- and school-aged children to be at particular risk of suffering from psychosocial stress during limited access to and closures of institutionalized daycare or elementary schools. For children, media consumption and physical activity seem to be important to regulate families' stress. For school-aged children, peer contact *via* digital means may offer a valuable resource to buffer stress.

Across the globe, countries are bracing themselves against new waves of the COVID-19 pandemic, with potential impediments for young families throughout the year 2021 and beyond. We hope that the current study informs scholars and policymakers on the manifold correlations of psychological well-being of parents and children during COVID-19 regulations and that it helps to provide targeted support to families.

## Data Availability Statement

The datasets presented in this study can be found in online repositories. The names of the repository/repositories and accession number(s) can be found below: https://osf.io/r84ca/.

## Ethics Statement

Ethical review and approval was not required for the study on human participants in accordance with the local legislation and institutional requirements. The patients/participants provided their written informed consent to participate in this study.

## Author Contributions

TT, RS, LS, and NS conceptualized the study and planned data acquisition. NA supported study conceptualization and data acquisition in Iran. TT, RS, and NS conducted the formal analysis and wrote the first draft of the manuscript. LS and NA commented on the manuscript. LS and TT curated the data. All authors contributed to the article and approved the submitted version.

## Conflict of Interest

The authors declare that the research was conducted in the absence of any commercial or financial relationships that could be construed as a potential conflict of interest.

## Publisher's Note

All claims expressed in this article are solely those of the authors and do not necessarily represent those of their affiliated organizations, or those of the publisher, the editors and the reviewers. Any product that may be evaluated in this article, or claim that may be made by its manufacturer, is not guaranteed or endorsed by the publisher.

## Footnotes

^1^Notably, such programs have been implemented successfully in some instances by local sport teams and federations (e.g., https://www.albaberlin.de/news/details/reaktion-auf-coronavirus-albas-taegliche-digitale-sportstunde-fuer-kinder-und-jugendliche/; https://www.sport.wales/beactivewales-campaign/beactivewales-keeping-young-people-active/).
