# Supplementary material for: Explaining Variation in Parents' and Their Children's Stress During COVID-19 Lockdowns
Source: Front Psychol. 2021 Sep 9;12:645266. doi: 10.3389/fpsyg.2021.645266 (PMC8458743; doi:10.3389/fpsyg.2021.645266)
Supplement: Supplementary file 1 [file Table_1.docx]

***Supplementary Material***

# Table S1. Schwartz’s cultural values

| **Scale** | **Value type** | **Motivational goal** |
| --- | --- | --- |
| Openness to change | Self-direction | Independent thought and action - choosing, creating, exploring |
|  | Stimulation | Excitement, novelty, and challenge in life |
|  | Hedonism | Pleasure and sense of gratification for oneself |
| Self-enhancement | Achievement | Personal success through demonstrating competence according to social standards |
|  | Power | Social status and prestige, control or dominance over people and resources |
| Self-transcendence | Universalism | Understanding, appreciation, tolerance, and protection for the welfare of all people and nature |
|  | Benevolence | Preservation and enhancement of the welfare of people with whom one is in frequent and personal contact |
| Conservation | Conformity | Restraints of actions, inclinations, and impulses likely to upset or harm others and violate social expectations or norms |
|  | Tradition | Respect, commitment, and acceptance the customs and ideas that traditional culture or religion provide |
|  | Security | Safety, harmony, and stability of society, or relationships and of self |

*Note.* This table is adopted from Schwartz (1992).

**Table S2. Predictors used in statistical models**

| *Label* | *Description* | *Coding Details* |
| --- | --- | --- |
| Age | Age of the participating parent | Age in years |
| Gender | Gender of the participating parent | Male vs. Female vs. Other |
| Home office | Mainly working from home | yes vs. no |
| Education | Highest degree in formal education of the participating parent | Secondary vs. Diploma vs. Bachelor vs. Master vs. PhD |
| Single parent | Caring children solitarily | yes vs. no |
| Parents’ stress^2^ | Stress item of the Parental Stress Scale (Zelman & Ferro, 2018) | Scale from 1 to 5 |
| Openness^1^ | Parents’ values on hedonism, stimulation, and self-direction | Scale from 1 to 6 |
| Self-enhancement^1^ | Parents’ values on power and achievement | Scale from 1 to 6 |
| Self-transcendence^1^ | Parents’ values on universalism and benevolence | Scale from 1 to 6 |
| Conservation^1^ | Parents’ values on conformity, tradition, security | Scale from 1 to 6 |
| Homeoffice | Working from home | yes vs. no |
| Community size | Population size of the participants’ community | <500 vs. <1,500 vs. <5,000 vs. <20,000 vs. <100,000 vs. <500,000 vs. <1,000,000 vs. <1,000,000 |
| Number of rooms^1^ | Number of bedrooms in the participants’ accommodation | Number of bedrooms |
| Garden | Access to a garden in the accommodation | yes vs. no |
| Restrictions | Presence of restrictions on leaving the accommodation due to Covid-19 | yes vs. no |
| Stringency score^1^ | Stringency score of the Oxford Covid-19 Government Response Tracker | 0 to 100 |
| Individualism score^1^ | Individualism score of Hofstede Insights | 0 to 100 |
| Constellation of children | Presence of both preschool-aged and school-aged children in the family | yes vs. no |
| Number of children^1^ | Number of children living in the families’ accommodation | Number of children |
| Parent Child Time^1^ | Daily time parents spend in direct interaction with their children | Number of hours |
| Change in parent child time^1^ | Change in the time spend in direct interaction compared to the time before the Covid-19 outbreak | Scale from 1 (time strongly decreased) to 7 (time strongly increased) |
| Homeschooling^1^ | Daily time parents spend to educate their children at home | Number of hours |
| Digital peer contact^1^ | Number of days per week children engage in digital peer contact | Scale from 1 to 7 |
| Parental support for digital peer contact^1^ | Parental support for their children’s digital contact with peers | Scale from 1 (not at all) to 7 (a lot) |
| Change in parental support for digital peer contact^1^ | Change in the parents’ support for digital peer contact as compared to the time before the Covid-19 outbreak | Scale from 1 (time strongly decreased) to 7 (time strongly increased) |
| Parental Support for Physical Activity^1^ | Parental support for their children’s physical activities | Scale from 1 (not at all) to 7 (a lot) |
| Change parental support for physical activity^1^ | Change in the parents’ support for physical activity as compared to the time before the Covid-19 outbreak | Scale from 1 (time strongly decreased) to 7 (time strongly increased) |
| Parental support for routines^1^ | Parental support for their children’s daily routines | Scale from 1 (not at all) to 7 (a lot) |
| Change in parental support for routines^1^ | Change in the parents’ support for daily routines as compared to the time before the Covid-19 outbreak | Scale from 1 (time strongly decreased) to 7 (time strongly increased) |
| Media consumption^1^ | Hours of children’s daily media consumption | Number of hours |
| Change in media consumption^1^ | Change in children’s media consumption as compared to the time before the Covid-19 outbreak | Scale from 1 (time strongly decreased) to 7 (time strongly increased) |
| Country | Country of residence | Name of country |

^1^scaled to M = 0 and SD = 1

^2^only included in models predicting children’s stress

**Table S3. Post-hoc tests for parental education for parents’ stress**

| *Comparison* | *z value* | *p* |
| --- | --- | --- |
| Secondary – A level | 0.166 | 1.000 |
| Secondary – Bachelor | 0.084 | 1.000 |
| Secondary – Master | 1.717 | .430 |
| Secondary – PhD | 3.384 | .006^*^ |
| A level – Bachelor | -0.121 | 1.000 |
| A level – Master | 0.724 | 1.000 |
| A level – PhD | 2.105 | .247 |
| Bachelor – Master | 1.890 | .352 |
| Bachelor – PhD | 3.597 | .003^*^ |
| Master - PhD | 2.648 | .065^†^ |

*Note*. ^*^*p* < .05; ^†^*p* < .1

**Table S4. Post-hoc tests for parental education for children’s stress**

| *Comparison* | *z value* | *p* |
| --- | --- | --- |
| Secondary – A level | 1.332 | 1.000 |
| Secondary – Bachelor | -1.067 | 1.000 |
| Secondary – Master | -1.344 | 1.000 |
| Secondary – PhD | -1.951 | .358 |
| A level – Bachelor | -2.007 | .358 |
| A level – Master | -2.154 | .281 |
| A level – PhD | -2.570 | .102 |
| Bachelor – Master | -0.181 | 1.000 |
| Bachelor – PhD | -1.245 | 1.000 |
| Master - PhD | -1.238 | 1.000 |

*Note*. ^*^*p* < .05; ^†^*p* < .1

**Table S5. Outcomes for German subsample**

|  | *Parents’ stress* | | | | |  | *Children’s stress* | | | | |
| --- | --- | --- | --- | --- | --- | --- | --- | --- | --- | --- | --- |
|  | *Preschool-aged children* | |  | *School-aged children* | |  | *Preschool-aged children* | |  | *School-aged children* | |
| *Full-null model comparison* | χ^2^(37) = 42.011, *p* = .027 | |  | χ2(37) = 55.085, *p* = .002 | |  | χ^2^(38) = 120.51, *p* < .001 | |  | χ^2^(38) = 74.01, *p* = .174 | |
|  | *estimate* | *S.E.* |  | estimate | *S.E.* |  | *estimate* | *S.E.* |  | *estimate* | *S.E.* |
| Age | -0.039 | 0.082 |  | -0.058 | 0.108 |  | 0.113 | 0.111 |  | 0.066 | 0.151 |
| Gender (ref: Male) |  |  |  |  |  |  |  |  |  |  |  |
| Female | 0.102 | 0.232 |  | 0.102 | 0.256 |  | -0.418 | 0.318 |  | -0.202 | 0.356 |
| Other | 1.146 | 0.975 |  | -0.481 | 1.002 |  | 0.322 | 1.339 |  | 0.821 | 1.395 |
| Home office | 0.085 | 0.161 |  | 0.110 | 0.188 |  | 0.098 | 0.220 |  | -0.029 | 0.262 |
| Education (ref: Secondary) |  |  |  |  |  |  |  |  |  |  |  |
| A level | **0.132*** | **0.447** |  | 0.160 | 1.006 |  | **0.643*** | **0.611** |  | -0.841 | 1.399 |
| Bachelor | **-0.190*** | **0.294** |  | -0.001 | 0.340 |  | **-0.071*** | **0.401** |  | 1.040 | 0.473 |
| Master | **0.351*** | **0.236** |  | 0.050 | 0.242 |  | **-0.441*** | **0.325** |  | 0.326 | 0.336 |
| PhD | **1.127*** | **0.357** |  | 0.169 | 0.435 |  | **-0.875*** | **0.505** |  | 0.369 | 0.606 |
| Single parent | 0.151 | 0.344 |  | 0.414 | 0.270 |  | 0.695 | 0.470 |  | -0.452 | 0.379 |
| Parents’ stress | -- | -- |  | -- | -- |  | **0.284*** | **0.115** |  | 0.309 | 0.141 |
| Openness | 0.009 | 0.092 |  | -0.125 | 0.103 |  | 0.122 | 0.126 |  | 0.027 | 0.144 |
| Self-enhancement | 0.042 | 0.090 |  | **0.218*** | **0.122** |  | -0.190 | 0.123 |  | -0.026 | 0.172 |
| Self-transcendence | 0.116 | 0.089 |  | 0.090 | 0.117 |  | **-0.479*** | **0.123** |  | 0.087 | 0.163 |
| Conservation | -0.084 | 0.090 |  | **-0.207*** | **0.103** |  | 0.194 | 0.123 |  | 0.280 | 0.146 |
| Community size (ref: < 500) |  |  |  |  |  |  |  |  |  |  |  |
| < 1500 | 0.416 | 0.803 |  | 0.234 | 0.591 |  | -1.397 | 1.098 |  | -0.516 | 0.823 |
| < 5000 | -0.053 | 0.693 |  | 0.034 | 0.523 |  | -1.505 | 0.947 |  | -0.892 | 0.727 |
| < 20.000 | 0.026 | 0.714 |  | -0.481 | 0.675 |  | -0.801 | 0.975 |  | 0.676 | 0.941 |
| < 100.000 | 0.512 | 0.707 |  | -0.377 | 0.649 |  | -0.837 | 0.967 |  | -1.426 | 0.905 |
| < 500.000 | 0.506 | 0.708 |  | 0.355 | 0.575 |  | -1.465 | 0.968 |  | -0.702 | 0.801 |
| < 1.000.000 | 0.208 | 0.710 |  | 0.085 | 0.571 |  | -1.521 | 0.970 |  | -0.482 | 0.795 |
| 1.000.000+ | 0.045 | 0.668 |  | 0.076 | 0.480 |  | -1.029 | 0.912 |  | -0.520 | 0.668 |
| Number of rooms | -0.025 | 0.103 |  | -0.148 | 0.130 |  | -0.113 | 0.140 |  | -0.481 | 0.181 |
| Garden | -0.299 | 0.194 |  | -0.231 | 0.255 |  | 0.182 | 0.267 |  | 0.008 | 0.356 |
| Restrictions | -0.134 | 0.155 |  | -0.148 | 0.177 |  | -0.295 | 0.212 |  | 0.345 | 0.246 |
| Constellation of children | **0.424*** | **0.234** |  | **0.737*** | **0.203** |  | -0.264 | 0.323 |  | -0.344 | 0.299 |
| Number of children | 0.064 | 0.098 |  | 0.086 | 0.106 |  | 0.188 | 0.134 |  | 0.131 | 0.148 |
| Parent-child time | 0.069 | 0.093 |  | 0.021 | 0.109 |  | 0.161 | 0.127 |  | -0.095 | 0.152 |
| Change in parent-child time | 0.137 | 0.091 |  | **0.224*** | **0.107** |  | **-0.304*** | **0.126** |  | -0.079 | 0.152 |
| Homeschooling | -0.122 | 0.073 |  | -0.013 | 0.102 |  | -0.039 | 0.101 |  | 0.083 | 0.142 |
| Digital peer contact | 0.029 | 0.097 |  | -0.045 | 0.088 |  | **0.247*** | **0.132** |  | 0.006 | 0.123 |
| Support for digital contact | 0.0002 | 0.087 |  | -0.166 | 0.105 |  | -0.004 | 0.119 |  | -0.174 | 0.148 |
| Change in support for digital contact | -0.008 | 0.093 |  | -0.033 | 0.100 |  | 0.106 | 0.128 |  | 0.052 | 0.140 |
| Support for physical activity | -0.181 | 0.114 |  | -0.119 | 0.111 |  | -0.029 | 0.157 |  | 0.079 | 0.155 |
| Change in support for physical activity | 0.044 | 0.098 |  | 0.101 | 0.108 |  | 0.127 | 0.134 |  | 0.148 | 0.151 |
| Support for routines | 0.061 | 0.096 |  | 0.059 | 0.112 |  | **-0.281*** | **0.132** |  | -0.201 | 0.156 |
| Change in support of routines | 0.027 | 0.097 |  | -0.029 | 0.115 |  | **0.360*** | **0.132** |  | 0.079 | 0.159 |
| Media consumption | -0.135 | 0.114 |  | -0.067 | 0.155 |  | 0.194 | 0.157 |  | 0.136 | 0.216 |
| Change in media consumption | **0.163*** | **0.092** |  | 0.035 | 0.107 |  | **0.234*** | **0.127** |  | 0.204 | 0.149 |
| Model determination (Marginal) | 0.248 | |  | 0.311 | |  | 0.335 | |  | -- | |

*Note*. S.E. = standard error; boldly printed estimates and standard errors marked with an asterisk indicate a significant pairwise comparison with *p* < .05. Model determination indicates marginal effects of all fixed effects.
